# Modifiable determinants of older adults’ physical activity and sedentary behavior in community and healthcare settings: a DE-PASS systematic review and meta-analysis

**DOI:** 10.1186/s11556-025-00373-y

**Published:** 2025-05-24

**Authors:** Simone Ciaccioni, Sofie Compernolle, Maren Lerfald, Federico Palumbo, Floriana Fadda, Ginevra Toma, Selcuk Akpinar, Katja Borodulin, Emine Caglar, Greet Cardon, Murat Cenk Celen, Joanna Cieślińska-Świder, Cristina Cortis, Andrea Di Credico, Murat Emirzeoğlu, Andrea Fusco, Daniel Gallardo Gómez, Linn Marita Hagen, Ayda Karaca, Mohammed Khudair, Marianna De Maio, Paul Jarle Mork, Livia Oddi, Kandianos Emmanouil Sakalidis, Petru Sandu, Sevil Turhan, Wei Wang, Melda Pelin Yargıç, Ekaterina Zotcheva, Laura Capranica, Ciaran MacDonncha, Linda Ernstsen

**Affiliations:** 1 Department of Education and Sport Sciences, Pegaso Telematic University, Naples, Italy; 2 Department of Movement, Human and Health Sciences, Foro Italico University of Rome, Rome, Italy; 3 Department of Movement and Sports Sciences - Physical Activity and Health, Ghent University, Ghent, Belgium; 4 Research Foundation Flanders, Brussels, Belgium; 5 Department of Public Health and Nursing, Faculty of Medicine and Health Sciences, Norwegian University of Science and Technology (NTNU), Trondheim, Norway; 6 Clinic of Medicine, St Olav’s University Hospital, Trondheim, Norway; 7 Department of Physical Education and Sport Sciences, Health Research Institute, University of Limerick, Limerick, Ireland; 8 Department of Public Health, Farmacy and Medicine, Sapienza University of Rome, Rome, Italy; 9 Department of Physiology and Pharmacology “V. Erspamer”, Sapienza University of Rome, Rome, Italy; 10 Department of Physical Education and Sport Teaching, Faculty of Sport Sciences, Nevşehir Hacı Bektaş Veli University, Nevşehir, Turkey; 11 Age Institute, Helsinki, Finland; 12 Department of Physical Education and Sport Teaching, Faculty of Sport Sciences, Hacettepe University, Ankara, Turkey; 13 Department of Biophysics, Faculty of Medicine, Bursa Uludag University, Bursa, Turkey; 14 Department of Physiotherapy of Movement Disorders and Sports Medicine, The Jerzy Kukuczka Academy of Physical Education in Katowice, Katowice, Poland; 15 Department of Human Sciences, Society and Health, University of Cassino and Lazio Meridionale, Viale dell’Università, Cassino, Italy; 16 Department of Medicine and Aging Sciences, University “G. d’Annunzio” of Chieti-Pescara, Chieti, Italy; 17 Department of Physiotherapy and Rehabilitation, Faculty of Health Sciences, Karadeniz Technical University, Trabzon, Turkey; 18 Andalusian Health Technology Assessment Area (AETSA), Progress and Health Public Foundation (FPS), Seville, Spain; 19 Division of Physical Activity and Health, Department of Recreation, Faculty of Sport Sciences, Hacettepe University, Ankara, Turkey; 20 School of Psychology, Cardiff University, Cardiff, United Kingdom; 21 Department of Information Engineering, Control and Management, Faculty of Information Engineering, Computer Science and Statistics, Sapienza University of Rome, Rome, Italy; 22 Department of Psychology, Northumbria University, Newcastle upon Tyne, United Kingdom; 23 National Institute of Public Health in Romania- Regional Public Health Center, Cluj-Napoca, Romania; 24 Department of Public Health, Karadeniz Technical University Medical Faculty, Trabzon, Turkey; 25 Department of Sport and Exercise Sciences, Durham University, Durham, United Kingdom; 26 Department of Physiology, Faculty of Medicine, Ankara Medipol University, Ankara, Turkey; 27 Norwegian National Centre for Ageing and Health, Vestfold Hospital Trust, Tønsberg, Norway

**Keywords:** Exercise, Inactive, Interventions, Randomized controlled trials, Controlled trials, Community-dwelling older adults, Seniors, Settings, Self-reported physical activity, Device-measured physical activity, Self-reported sedentary behaviour, Device-measured sedentary time

## Abstract

**Objectives:**

To identify the modifiable determinants targeted in interventions involving older adults, and to determine which of these interventions effectively increased physical activity (PA) and/or reduced sedentary behaviour (SB). Additionally, to explore whether the effects of these interventions vary based on the implementation setting.

**Methods:**

A search of randomized controlled trials (RCTs) and controlled trials (CTs) was performed in Medline, APA PsycArticles, SPORTDiscus, and Web of Science. Risk of bias assessment was performed with Cochrane’s tool. Modifiable determinants were narratively synthesized, and random-effects models were performed to meta-analyse studies reporting device-measured physical activity or sedentary behaviour. Moderator analyses were performed to investigate the role of implementation setting. Standardized between-group mean difference (SMD) with 95% confidence interval (CI) was used to indicate effect sizes.

**Results:**

From 31,727 individual records, 52 eligible studies published between 2012–2022 were identified, 30 and 22 studies from community and health care settings, respectively. Determinants within the category physical health and wellbeing (*n* = 23) were most frequently reported while only one study reported determinants within a social or cultural context. Eighteen studies were included in the meta-analysis. Interventions targeting physical health and wellbeing revealed an increase in steps (SMD = 0.46; 95%CI: 0.15 to 0.77) and minutes of moderate-to-vigorous intensity physical activity (SMD = 0.41; 95%CI: 0.19 to 0.64) among intervention participants compared to controls, whereas interventions targeting psychological or behavioural determinants showed no between-group differences in steps (SMD = 0.10; 95%CI: -0.12 to 0.32) and moderate-to-vigorous intensity physical activity (SMD = 0.26; 95%CI: -0.24 to -0.75). Interventions targeting physical health and wellbeing showed significant heterogeneity (*p* < 0.0001; I^2^ = 73.10%). Subgroup analyses showed a significant effect on device-measured physical activity for the eight community-based interventions (SMD = 0.42; 95%CI: 0.07 to 0.77), while no significant effect was found for the eight studies performed in healthcare settings (SMD = 0.26; 95%CI; -0.10 to 0.62).

**Conclusion:**

Interventions targeting physical health and wellbeing may increase PA in older adults, with community-based studies appearing more effective than studies in healthcare settings. The significant heterogeneity of study findings indicates that further research is needed to fully understand the influence of PA and SB determinants across settings, particularly those related to psychological, behavioural, social, and cultural factors.

**Systematic review registration:**

PROSPERO: CRD42022287606.

**Supplementary Information:**

The online version contains supplementary material available at 10.1186/s11556-025-00373-y.

## Introduction

Physical inactivity and excessive sedentary behaviour (SB) increases the risk of premature death and disease and adverse health conditions, including cardiovascular diseases, cancer, chronic respiratory diseases and diabetes [1]. A recent study, which analyzed self-reported PA data from 507 population-based studies, estimated that the global prevalence of insufficient PA among older adults is 43.5% [2]. When considering population-based studies providing device-measured PA findings, less than 10% of the older adults adheres to the PA guidelines [3–5]. Moreover, despite an insufficient evidence to define a threshold for sedentary behaviour (SB) [6], to prevent age-related falls, osteoporosis, and decline of functional ability and fitness (e.g., strength, balance, flexibility) older adults would benefit from sitting less, breaking up their sitting time, and moving more [7, 8].

A modifiable determinant for physical PA and/or sedentary SB refers to any factor that can be altered or influenced through interventions to promote healthier behaviors. For example, enhancing access to recreational facilities and encouraging active transportation can boost PA, while increasing awareness of the negative health effect and fostering supportive social environments can reduce SB. However, there are still gaps in understanding the most effective strategies for various populations and contexts, especially in older adults. For instance, while many interventions have been designed to modify PA and SB, their effectiveness varies widely. To develop interventions for increasing PA [9] and reducing SB [7, 10] it is necessary to identify non-modifiable (e.g., genetics, age) and modifiable (e.g., muscle strength, sport facilities, intrinsic motivation, social support) determinants [11], the latter needing a public health and policy special focus [11, 12]. The European Determinants of Diet and Physical Activity (DEDIPAC) Knowledge Hub was the first action taken by the ‘Healthy Diet for a Healthy Life’ European Joint Programming Initiative [13]. DEDIPAC aimed to provide insight into the determinants of diet, PA and SB across the life course. Analyses of systematic literature reviews revealed several knowledge gaps regarding behavioural, biological, physical-environmental, policy, psychological, socio-cultural, and socio-economic PA and SB determinants [13]. Further, methodological issues such as lack of studies with device-measured PA and SB and a predominance of cross-sectional studies were also reported [13]. A DEDIPAC study on sedentary time and PA surveillance of older adults and obese individuals across four European countries concluded that there is an urgent need to provide new knowledge on modifiable PA and SB determinants in various settings to reduce the prevalence of unhealthy behaviours in these populations [14].

In the last four years, the European COST Action CA19101 “Determinants of Physical Activities in Setting (DE-PASS) focused on identifying, understanding and measuring modifiable determinants which promote, maintain or inhibit PA and SB across the lifespan and in different settings (https://depass.eu/) [15–18]. The influence of settings is critical as context shapes intervention effectiveness, influencing factors such as accessibility, motivation, and support. For instance, whilst community settings typically focus on preventive measures and address broad social determinants of health, healthcare settings generally prioritize direct medical care and individual treatments. However, context is seldom taken into account when intervention studies are planned [19]. To explore and address a number of different health behaviours including PA and SB, ecological models have been developed [20, 21]. Despite the growing recognition of these multiple levels of influences, to date no systematic literature reviews with or without meta-analyses have identified the most commonly studied modifiable determinants of PA and SB from randomized controlled trials (RCTs) or controlled trials (CTs) in older adults. Furthermore, there is a need to assess the effectiveness of these determinants in relation to different settings. Moreover, device-based measurements are particularly sensitive to changes in both PA and SB [22], suggesting that meta-analyses should ideally separate studies using self-reported data from those using device-based measurements to maintain consistency and accuracy of the results. In considering the variability in the existing literature, an exploratory approach without specific hypotheses is needed.

Therefore, the main objectives of the present DE-PASS Systematic Review and Meta-analysis (SRMA) study on RCTs and CTs including older adults are to identify the modifiable determinants targeted in interventions involving older adults, and to determine which of these interventions effectively increased PA and/or reduced SB. Additionally, to explore whether the effects of these interventions vary based on the implementation setting (community versus healthcare).

## Material and methods

This study was conducted according to the updated Preferred Reporting Items for Systematic Review and Meta-Analysis (PRISMA 2020) guidelines [23]. The protocol of this review was registered in the International Prospective Register of Systematic Reviews (PROSPERO ID: CRD42022287606).

### Eligibility criteria

To ensure clarity, consistency, and relevance in addressing the research objectives, the research methodology integrated the following five elements: Population, Intervention, Comparison, Outcomes Context (PICOC).

### Population (old* OR elder* OR adult* OR aged people OR ageing* OR senior* OR veteran* OR mature*…)

To be eligible for inclusion studies had to include adults aged ≥ 65 years. However, to reduce study heterogeneity exclusion criteria encompassed studies targeting individuals 1) diagnosed with dementia, encompassing all dementia subtypes (e.g., Lewy body dementia, Alzheimer's disease, Vascular dementia, Mixed dementia, Frontotemporal lobe dementia); 2) currently hospitalized due to surgical or medical treatments (e.g., cancer treatment); 3) receiving terminal or palliative care; and 4) undergoing pre- and post-operative orthopaedic investigations of the spine or lower extremities. In fact, PA and SB in persons with dementia is highly dependent on support and influence from family and professional caregivers [24], further, specific medical diagnoses and medical treatment (e.g., of the musculoskeletal system) may cause pain, discomfort, and the need for practical assistance and support provided from others.

### Intervention (Physical activ* OR exercise OR sport* OR play OR recreation OR training…)

Interventions were considered for inclusion when their primary outcome was metrics of PA (e.g., total PA, leisure-time PA, or MVPA), and/or SB (e.g., sedentary time) obtained either through device-based measurements (e.g., pedometers, accelerometers, etc.), or self-reported data collected via validated questionnaires. The included studies had to report pre- and post-intervention measurements of determinants, as well as of PA and/or SB within the framework of controlled intervention trial designs. Therefore, only RCTs and CTs were included, as these trials sit at the top of the research hierarchy and are considered the most appropriate study designs for developing a best evidence statement.

### Comparison (old* OR elder* OR adult* OR aged people OR ageing* OR senior* OR veteran* OR mature*…)

Considering the PA and SB self-report and device-based measurement methods, matching of control groups, or other active intervention groups to the experimental groups for the selected studies were examined. In addition, a comparative synthesis of findings across outcomes resulting from the respective measurement methods, with special attention to the settings of the selected modifiable determinants was implemented.

### Outcomes (Physical activ* OR exercise OR sedentar* OR screen time OR computer use OR determinant*…)

PA and SB are the main quantitative outcome measures targeted in the present research. PA is defined as any bodily movement produced by skeletal muscles that requires energy expenditure, thus including any modality of movement at any intensity [25]. PA are also categorised as sedentary, light, moderate and vigorous intensity PA and SB as any waking behaviour characterised by an energy expenditure of 1.5 METs or lower while sitting, reclining or lying, while SB [26]. Measurement techniques for PA and SB encompassed validated self-report (e.g., questionnaires, diaries, recall) and device-based (e.g., accelerometers, pedometers) methods [22]. In studies where both device-based and self-report measures are reported, the data for both measurement methods will be extracted and analysed separately. For this review, the categories of modifiable determinants were based on a framework from the DEDIPAC consortium, consisting in six clusters (i.e., Physical Health and Wellbeing, Social and Cultural Context, Built and Natural Environment, Psychology and Behaviour, Politics and Economics, and Institutional and Home Settings) obtained through an international transdisciplinary consensus framework for the study of determinants, research priorities and policies on sedentary behaviour across the life course [27].

### Context (mediator* OR moderator* OR exposure*…)

Settings were retrospectively considered in this systematic review as a contextual factor potentially influencing the association between modifiable determinants and PA or SB of older adults. To examine how these environments moderated the effectiveness of interventions, studies were categorized based on their primary setting. This categorization allowed for the evaluation of context-specific variations in determinants, ensuring that the findings reflect the potential impact of different settings on PA and SB outcomes.

### Information sources and search strategy

Based on the collaboration of the DE-PASS Team and with the support of professional librarians (K.H., L.S., T.Z.), a systematic literature search was conducted in September 2022. Studies published since 2012 have been included as this timeframe is sufficient to capture relevant and contemporary published research, it aligns with important WHO publications related to physical activity and health, and with the publications of key guidelines and SLRs on the topic of physical activity and the older adult [28–31]. To ensure the inclusion of the most recent articles, alert notifications of new publications were activated until August 2024. In addition, the snowballing technique was applied to the reference lists of the included articles to identify any possible missed scientific contribution. The search was performed in MEDLINE via PubMed, APA PsycArticles and SPORTDiscus via EBSCOhost, and Web of Science (Core Collection). Adapted to the specific features of each database, the search strategy included a combination of subject heading terms and keywords related to the outcome variables (i.e., PA and SB), the study designs (i.e., CTs and RCTs), the determinants addressed by the intervention, the target older adult population, and the measurement methods for PA and SB. The search was limited to peer-reviewed academic articles published within the past decade and written in languages in which members of the review team have an advanced level of proficiency (i.e., Dutch, French, Greek, Italian, Norwegian, Polish, Romanian, Spanish, Turkish). Details on the search strategy –– are presented in Supplementary File 1. Results of the search were uploaded to the online tool Covidence (Veritas Health Innovation, Melbourne, Australia) supporting the screening, study selection, data extraction of systematic literature reviews (SLRs), and duplicates removal.

### Selection and data collection processes

The screening process was conducted by a large multidisciplinary review team (*n* = 28) of DE-PASS members. To ensure a homogenous procedural proficiency and agreement among the reviewers, prior to screening initiation the core group (S.Ci., L.E., S.Co., M.L., F.P., G.T., F.F.) provided several workshops and tutorials on various aspects, including inclusion and exclusion criteria, data extraction forms and risk of bias assessment tools. Then, the review team was divided into pairs, and Covidence facilitated a random and equitable distribution of studies. Utilizing a pre-tested decision tree with inclusion/exclusion criteria outlined in Supplementary File 2, each reviewer pair independently evaluated the eligibility of the assigned studies based on the titles and abstracts (stage 1), followed by full-text examination (stage 2). Any discrepancies were resolved by a third reviewer from the core group. The reviewers identified if the same studies were included in different reviews by comparing authors, demographics, ethical committee approval, intervention content and design, sample size, study locations, and settings [32]. At the end of Stage 2, in case of duplicates the inclusion was based on the study reporting the highest number of measurement timepoints or the longest follow-up periods [33].

### Data items and extraction

The first author (SCi) drafted a data extraction form, which underwent pilot-testing by the core group. Subsequently, the included studies were distributed among review pairs from the multidisciplinary review team for an independent data extraction. To ensure accuracy and consistency, a consensus procedure was applied through bilateral online meetings to resolve any disagreements by comparing the results, discussing the possible differences and diverse interpretations, retrieving the relative source of discrepancies, and reaching an agreement. If consensus was not reached, a third reviewer from the core group was consulted to provide an independent evaluation and make a final decision. Data extraction included source description (e.g. author, year of publication, and country of publication), sample characteristics (e.g. sample size, demographic characteristics), intervention and control condition description (e.g. intervention[s] and control group activity[-ies] including modifiable determinants, intervention duration, implementation setting(s]), and study measures (e.g. PA and/or SB, self-reported vs device-measured). Additionally, information on the study outcomes, both in terms of determinants and PA/SB, was extracted. In case of missing data or need of clarifications, the reviewers contacted the corresponding author of the respective studies allowing a reasonable period of two weeks for complete reporting before finalizing data extraction.

### Risk of bias

The modified version of Cochrane’s Risk of Bias 2 tool for randomized trials (RoB 2) [34] was used for the assessment of risk of bias. To ensure familiarity with the studies, the same two independent reviewers who extracted the data also performed the risk of bias assessment using forms based on templates aligned with the RoB 2 tool. In case of conflicts and to ascertain the correctness of assessment, the two reviewers performed a consensus procedure.

### Data synthesis and analysis

**Fig. 1:**
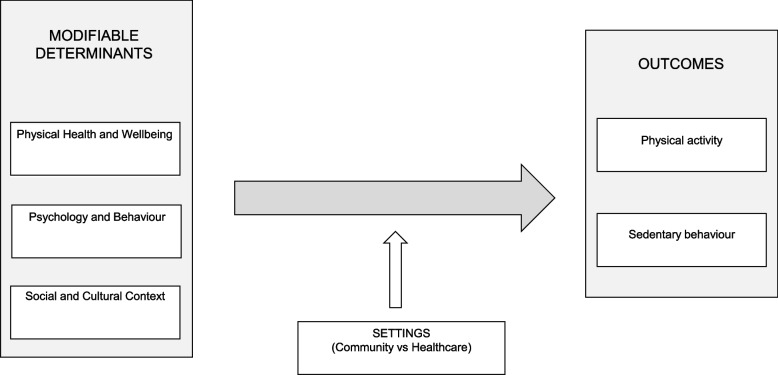
Conceptual model of the relationship between groups of modifiable determinants, settings and PA/SB outcomes

The extracted data of all included studies were narratively synthesized with a special focus on the modifiable determinants of PA and SB. Modifiable determinants were categorized based on a previously published DEDIPAC study [27]. The settings where the intervention took place were classified into community and healthcare settings. A conceptual model outlining the relationships between groups of determinants, settings and outcomes is illustrated in Fig. 1. Each study was summarized based on the outcome measure, and *P* values and confidence intervals (CIs) were analysed to determine the significant effects of the intervention. A two-tailed *p*-value of less than 0.05 was considered statistically significant. Between-group interactions were analysed, and the presence/absence of statistical significance and the direction (increase/decrease) of the outcome changes have been highlighted (see =, ↑ and ↓ symbols in Table 1). Given our priority to evaluate the effects of interventions on determinants of PA and SB through a meta-analysis of studies using device-measures of PA/PB and due to article length considerations, the included studies were narratively synthesized without applying the recently developed Synthesis Without Meta-analysis (SWiM) methodology [35] for self-reported data.

#### Meta-analyses

Meta-analyses were conducted exclusively for studies using device-based measurements of PA and/or SB. This approach was chosen to strengthen the validity, as devices like accelerometers or pedometers provide precise data and reduce the risk of recall and reporting biases. Meta-analyses were conducted using R, version 4.3.2 (R Foundation for Statistical Computing) and included only studies reporting means or standard deviations and outcome data for device-measured PA (steps/day and MVPA/min/day) and SB (sedentary time/min/day) at the end of the intervention. Given the continuous nature of the outcome variables, the standardized between-group mean difference (SMD) with corresponding 95% confidence interval (CI) were used as effect sizes and was calculated by comparing the outcome variables between the intervention and control groups at post-test. To re-express the SMD in steps per day, and minutes of MVPA/SB, we multiplied the pooled SMD by the pooled standard deviation (SD), yielding an estimate of the absolute difference in steps per day and minutes of MVPA/SB between the groups [36]. We assumed that there were no significant differences in PA and SB between the intervention and control groups at baseline, as the included studies were RCTs. Subsequently, random effects models (using the Hartung-Knapp method) were performed to calculate pooled estimates due to the expected heterogeneity. Our conceptual model initially proposed conducting three separate random effects models for each outcome variable (steps/day, MVPA/min/day, sedentary time/min/day): one for interventions focusing on physical health and wellbeing determinants, one for interventions targeting psychological and behavioural determinants, and one for interventions focusing on social and cultural context. However, due to the inclusion of only a single study targeting the social and cultural context, we were unable to conduct random effects models for this category. Forest plots were generated to visualize the results of the meta-analyses.

#### Test of heterogeneity and moderation analyses

The existence of heterogeneity was assessed using the Cochran’s Q test, and the Higgin and Thompson’s I^2^ statistics to determine whether moderation and subgroup analyses would be meaningful. A Q-value with a *p* ≤ 0.05 was considered indicative of significant heterogeneity, while I^2^ values of 25%, 50% and 75% were considered indicative of low, moderate and high heterogeneity, respectively [37]. If moderate or high heterogeneity was present, moderator analyses using a mixed-effects model with maximum likelihood estimation were conducted to test whether the heterogeneity could be explained by differences in settings (healthcare vs community).

#### Sensitivity analyses and publication bias check

Sensitivity analyses were carried out to investigate the robustness of the statistical analysis. Concretely, main analyses were repeated without potential outliers, which were defined as studies whose 95% CI did not overlap with the aggregated effect size's 95% CI [38, 39]. Main analyses were also repeated without low-quality studies. Studies were considered to be of low quality if their overall risk of bias, as assessed using the revised version of the RoB 2 tool, was determined to be high [40] (see Supplementary File 6). The presence of publication bias was assessed using visual examination of the funnel plot symmetry and by interpreting the results of the Egger’s regression test.

## Results

### Study selection

**Fig. 2:**
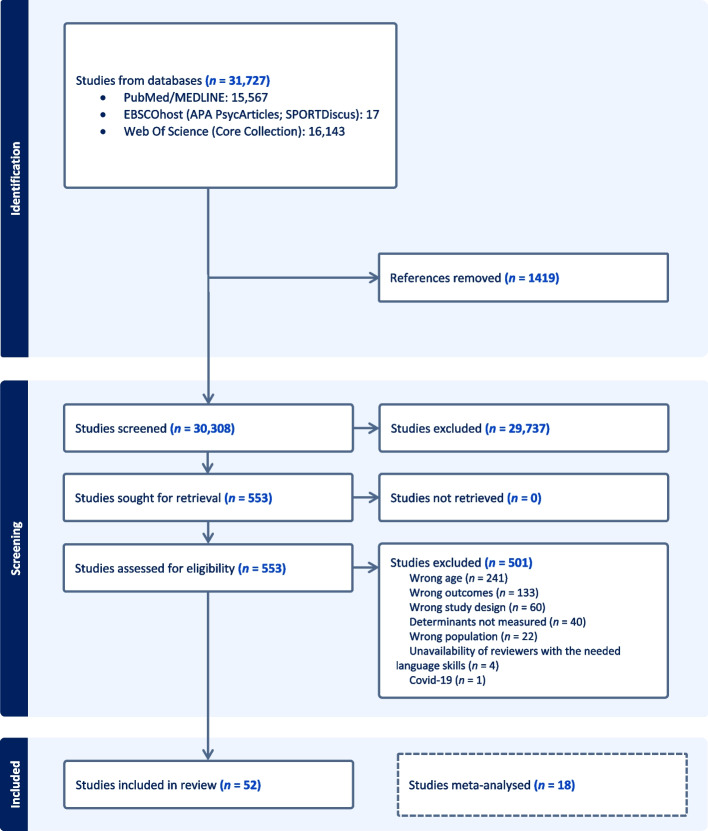
PRISMA flow diagram showing the systematic literature review process

Overall, 31,727 records were identified after removing duplicate results, 30,308 studies were entered in the screening process. The Fig. 2 shows the PRISMA flow diagram [23]: title and abstract screening resulted in 553 full-text articles, of which 52 RCTs [41–92] were included in the narrative synthesis. None of them contained studies that we would consider for further inclusion. Moreover, 18 studies using device-based measurements of PA and/or SB were included in the meta-analyses [41, 44, 49, 50, 52, 56, 60, 61, 66, 68, 71, 72, 76, 77, 80, 81, 85, 92]. No CTs were included. Included studies were published between January 2012 and September 2022.

### Study characteristics

The characteristics of the included studies are summarized in Supplementary Table 1. A total number of 9,112 older individuals (58% female), participated in the included RCTs. Number of study participants in individual studies ranged from 26 [56] to 616 [67] participants. The RCTs were conducted in America (*n* = 18), Asia (*n* = 7), Europe (*n* = 15) and Oceania (*n* = 12).

### Determinants

Overall, 44 unique modifiable determinants were identified in the 52 RCTs. Following a DEDIPAC consensus framework [27] (see Fig. 1), these modifiable determinants were classified as physical health and wellbeing (*n* = 31), psychology and behaviour (*n* = 12) and social and cultural context (*n* = 1). For physical health and wellbeing, the most frequently targeted determinants were physical functioning (*n* = 25 [41–50, 52, 60, 61, 64, 67–70, 82–84, 86, 88–90]), quality of life (*n* = 27 [44, 45, 47, 49, 53–55, 58–60, 62, 64–67, 69, 71–76, 79, 80, 86, 89, 90]), and body composition (*n* = 12 [41, 43, 44, 51, 59, 63, 65, 66, 73, 77, 88, 92]). In the psychology and behaviour category, self-efficacy was the most frequently targeted determinant (*n* = 13 [46, 47, 50–52, 55, 59, 60, 65, 78, 79, 85, 90]), while social support (*n* = 1 [86]) was the sole determinant investigated within the social and cultural context category. Detailed information about the modifiable determinants and the impact of interventions on these determinants is provided in Table 1.

**Table 1:** Summary of randomised controlled trials on older adults’ physical activity- and sedentary behaviour-related interventions

[N]	1 st Author-Country	Year^#^	SS (F)	Age: year^*^ Setting^§^	Population	Intervention	Comparison	Time: weeks	Determinants	PA/SB measures	Outcomes
[41]	Arrieta H-Spain	2019	*N* = 112 (75%)	80.4 (± 8.6) Healthcare	Long-term nursing home residents	IG: multicomponent exercise	CG: routine LIPA exercise	26	FF, Body composition	DM-PA	IG vs CG: = PA, ↑FF, = body composition
[42]	Baker BS-USA	2021	*N* = 46 (76%)	68.1 (± 7.4) Community	Community-dwelling older adults	IG1: resistance training program	CG1: no structured PA CG2: walking	8	FF, sleep	SR-PA	IG1 (vs CG1 & CG2): ↑auxiliary PA, ↑FF, ↑sleep
[43]	Barone Gibbs B-USA	2017	*N* = 38 (71%)	68 (± 7) Community	Community-dwelling older adults	IG: reaching ≥ 150’ of MVPA/week	CG: reducing 1 h sedentary time/day	12	BP, FF, Weight	DM-PA; SR-PA; SR-SB	IG vs CG: ↑DB MVPA, = SB, = BP, = FF, = weight
[44]	Brickwood KJ-Australia	2021	*N* = 117 (64%)	72.4 (± 6.5) Community	Community-dwelling older adults	IG1: PA tracking IG2: counselling	CG: usual care	52	BMI, BP, FF, QoL	DM-PA; SR-PA	IG1 vs CG: = PA, ↑BP, = BMI, = FF, = QoL IG2 vs CG: = PA, = BP. = BMI, ↑FF, ↑QoL
[45]	Brovold T-Norway	2013	*N* = 115 (61%)	78.0 (± 5.2) Community	Community-dwelling older adults	IG: HIPA aerobic interval exercise	CG: home-based LIPA exercise	13	FF, QoL	SR-PA	IG vs CG: = PA, ↑FF, = QoL
[46]	Callahan LF-USA	2014	*N* = 339 (83%)	68.8 (± 1.3) Community	Community-dwelling older adults	IG: theory-based lifestyle program	CG: wait list	20	ADL, arthritis, FF, self-efficacy, outcome expectations	SR-PA	IG vs CG: ↑PA, = ADL, ↑FF, ↑endurance, = arthritis symptoms, = self-efficacy, = outcomes expectations
[47]	Cameron-T. HL-Australia	2014	*N* = 221 (47%)	65.8 (± 9.4) Healthcare	Outpatients with COPD	IG: supervised aerobics & strength	CG: self-management education in group	6	Exercise capacity, FF, QoL, self-efficacy, stage of change	SR-PA	IG vs CG: ↑PA, ↑exercise capacity, = FF, QoL, = exercise self-efficacy, = stage of change
[48]	Chan MLT-China	2022	*N* = 124 (86%)	77.7 (± 7.4) Community	Community-dwelling older adults	IG: MIPA stepping exercise program	CG: health-education program	12	Fatigue, FF, mood, frailty	SR-PA; SR-SB	IG vs CG: ↑PA, ↓fatigue, = FF, ↓frailty,
[49]	Cheng SWM-Australia	2022	*N* = 65 (50%)	73.5 (± 9.5)Healthcare	Outpatients with COPD	IG: guided behaviour change education	CG: health status monitoring via phone	6	Depression, Anxiety, FF, QoL	DM-PA; DM-SB	IG vs CG: = SB, ↑MVPA, ↑step count, ↓depression
[50]	Clark IN-Australia	2017	*N* = 56 (21%)	68,2 (± 6.5)Healthcare	Cardiac rehabilitation outpatients	IG: self-selected music & usual care	CG: usual care	5	Anthropometry, FF, self-efficacy	DM-PA; DM-SB	IG vs CG: = PA, = SB, = anthropometry, = FF, = self-efficacy
[51]	Clemson L-Australia	2012	*N* = 317 (55%)	82.8 (± 4.5) Community	Community-dwelling older adults	IG1: multicomponent IG2: balance/strength	CG: gentle sham exercise	26	ADL, function, BMI, balance, strength, health status, self-efficacy	SR-PA	IG1 vs CG: ↑ADL, ↑balance, ↑strength, ↑function, ↑PA, ↑health status, = BMI, ↑balance self-efficacy, ↓falls IG2 vs CG: ↑balance, ↓BMI, ↑function, ↑balance self-efficacy, = falls
[52]	Cox KL-Australia	2021	*N* = 52 (64%)	70.1 (± 6.4) Community	Community-dwelling older adults	IG: home-based MIPA goal setting with peers	CG: home-based MIPA standard education	26	FF, self-efficacy, goal performance	DM-PA; SR-PA	IG & CG: ↑PA, ↑FF, ↑goal performance IG vs CG: no between-group differences
[53]	Dale MT-Australia	2014	*N* = 35 (0%)	71 (± 6.5) Healthcare	Outpatients with respiratory diseases	IG: supervised aerobic exercise	CG: usual care	8	Exercise capacity, QoL	DM-PA	IG vs CG: ↑PA, ↑exercise capacity, ↑QoL
[54]	Delbaere K-Australia	2021	*N* = 503 (67%)	77.5 (± 5.5) Community	Community-dwelling older adults	IG: balance e-exercise & health education	CG: health education	104	Wellbeing, QoL, balance, fall rate	DM-PA; SR-PA	IG vs CG: = PA, ↓fall rate (at 24 months), = wellbeing, = QoL, ↑balance
[55]	DeRoos P-Netherlands	2018	*N* = 52 (66%)	70.2 (± 9.5) Healthcare	Outpatients with COPD	IG: exercise & home walking & education	CG: usual care	10	Exercise capacity, self-efficacy, QoL	DM-PA	IG vs CG: ↑PA, ↑exercise capacity, = QoL, = self-efficacy
[56]	Ehrari H -Denmark	2020	*N* = 26 (73%)	83.5 (± 7.1) Community	Community-dwelling older adults	IG: multicomponent games-based program	CG: usual daily activities	12	Strength, endurance	DM-PA	IG vs CG: = PA, = strength, = endurance
[57]	Gallo E-USA	2018	*N* = 69 (27%)	78.4 (± 8,9) Community	Community-dwelling older adults	IG: MVPA home exercise & consults	CG: usual care	26	Balance	SR-PA	IG vs CG: ↑PA, ↑balance
[58]	Gaunaurd IA-USA	2014	*N* = 72 (NA)	68.5 (± 0.7) Healthcare	Outpatients with pulmonary fibrosis	IG: strengthening, aerobics & education	CG: usual daily activities	12	QoL	SR-PA	IG vs CG: ↑PA, ↑QoL
[59]	Herring LY-UK	2021	*N* = 291 (84%)	66.5 (± 9.7) Healthcare	Outpatients with coronary heart disease	IG: PA education & walking advises	CG: usual care & health advice	2	Anxiety, BMI, depression, self-efficacy, QoL	DM-PA; SR-PA; DM-SB; SR-SB	IG vs CG: = PA, ↓SB, = anxiety, = BMI, = depression, = self-efficacy, = QoL
[60]	Hinrichs T-Germany	2016	*N* = 209 (74%)	79.8 (± 5.2) Healthcare	Chronically ill mobility-limited outpatients	IG: multicomponent program & consults	CG: PA promotion & consults	12	Self-efficacy, FF, QoL	DM-PA	IG vs CG: = PA, = self-efficacy, = QoL
[61]	Hirase T-Japan	2018	*N* = 76 (76%)	78.3 (± 6.1) Community	Community-dwelling older adults	IG: strength/balance & pedometer & diary	CG: strength/balance training only	12	Pain, psychological status, FF	DM-PA	IG vs CG: ↑PA (steps), = pain, = psychological status, = FF
[62]	Iliffe S-UK	2014	*N* = 1256 (62%)	73 (NA) Community	Community-dwelling older adults	IG1: group exercise IG2: home exercise	CG: usual care	24	Fall rate, balance confidence, QoL	SR-PA	IG1 vs CG: ↑PA, ↓fall rate, ↑balance confidence, = QoL | IG2 vs CG: = PA, = QoL
[63]	Jancey J-Australia	2017	*N* = 280 (75%)	72 (± 5.2) Community	Retirement village residents	IG: PA and nutrition promotion & support	CG: no PA programs	26	BMI, BP	SR-PA; SR-SB	IG vs CG: ↑PA, = anthropometry, = BP
[64]	Kerr J-USA	2018	*N* = 307 (72%)	81.9 (± 5.9) Community	Retirement village residents	IG: peers-assisted walking & pedometer	CG: successful aging education & calls	52	BP, FF, QoL, stress, FoF	DM-PA	IG vs CG: ↑PA, ↓BP, = FF, = QoL, = stress, = FoF
[65]	Khunti K-UK	2021	*N* = 353 (54%)	67.8 (± 9) Healthcare	Outpatients with multimorbidity	IG: group-based PA self-management	CG: usual care	52	QoL, depression, self-efficacy, BMI, BP	DM-PA; DM-SB	IG vs CG: ↓PA, ↓QoL, = depression, = self-efficacy, = BMI, = BP
[66]	Ko FW-China	2021	*N* = 136 (3%)	75 (± 6.7) Healthcare	Outpatients with COPD	IG: physiotherapist supervised training	CG: usual care	52	BMI, Exercise capacity, QoL	DM-PA; DM-SB	IG vs CG: = PA, = BMI, = exercise capacity, ↑QoL
[67]	Lee HC-Taiwan	2013	*N* = 616 (55%)	75.7 (± 7.1) Community	Community-dwelling older adults	IG: multicomponent exercise & education	CG: health promotion only	8	Falls, FF, QoL, depression	SR-PA	IG vs CG: ↑PA, ↑FF, ↑QoL, ↓depression
[68]	Mackey DC-Canada	2019	*N* = 58 (0%)	71.9 (± 6.6) Community	Community-dwelling older adults	IG: group & individual support & pedometer	CG: waitlist	12	FF	SR-PA; DM-PA, DM-SB	IG vs CG: ↑PA, = FF
[69]	McDermott MM-USA	2018	*N* = 200 (53%)	70.2(± 10.4) Healthcare	Outpatients with PAD	IG: home exercise & wearable & calls	CG: usual care	39	Pain, FF, QoL	DM-PA; SR-PA	IG vs CG: = PA, ↑pain, = FF, = QoL
[70]	McMahon SK-USA	2016	*N* = 30 (93%)	83.6 (± 0.9) Community	Community-dwelling older adults	IG: mHealth app & small group exercise	CG: health information & small group exercise	8	FF, social support, self-regulation, goal attainment, change readiness	DM-PA; SR-PA	IG vs CG: ↑PA, ↑FF, ↑social support, ↑self-regulation, = goal attainment, ↑readiness to change
[71]	Mendoza L-Chile	2015	*N* = 102 (39%)	68.6 (± 8.5) Healthcare	Outpatients with COPD	IG: pedometer & PA diary	CG: PA diary & PA counselling	13	QoL, exercise capacity	DM-PA	IG vs CG: ↑PA, ↑QoL, ↑exercise capacity
[72]	Morén C-Sweden	2016	*N* = 88 (53%)	71 (± 8.7) Healthcare	Outpatients with TIA	IG: individualized PA prescription	CG: usual care & health information	26	Exercise capacity, QoL	DM-PA; DM-SB	IG vs CG: = PA, ↑exercise capacity, = QoL
[73]	Morey MC-UK	2012	*N* = 302 (3%)	67.4 (± 6.2) Healthcare	Outpatients with prediabetes mellitus	IG: aerobics and strength exercise	CG: usual care & PA advices	52	BMI, QoL, exercise capacity	SR-PA	IG vs CG: ↑PA, = BMI, = QoL, = exercise capacity
[74]	Moy ML-USA	2016	*N* = 238 (6%)	67 (± 9) Healthcare	Outpatients with COPD	IG: walking & pedometer & website	CG: waitlist	8	QoL	DM-PA	IG vs CG: ↑PA (at 4 months), = PA (at 12 months), = QoL
[75]	Nolan CM-UK	2017	*N* = 152 (28%)	68 (± 9) Healthcare	Outpatients with COPD	IG: pedometer & COPD rehabilitation	CG: pulmonary rehabilitation program	26	Exercise capacity, QoL	DM-PA	IG vs CG: = PA, = exercise capacity, = QoL
[76]	Oliveira JS-Australia	2019	*N* = 131 (47%)	71 (± 6.5) Community	Community-dwelling older adults	IG: expert coaching, pedometer, brochure	CG: fall prevention brochure	26	Falls, QoL, FoF, mood, mobility, goal attainment	DM-PA	IG vs CG: = PA, = falls, = QoL, = FoF, = mood, = mobility, ↑goal attainment (at 6 months, not at 12 months)
[77]	Owari Y-Japan	2019	*N* = 80 (70%)	72.7 (± 5.5) Community	Community-dwelling older adults	IG: health promotion lectures & brochures	CG: no activities nor intervention	52	BMI, sleep, stress	DM-PA; DM-SB	IG vs CG: = PA, ↓SB, = BMI, = sleep, = stress
[78]	Park YH-Korea	2014	*N* = 43 (79%)	77.7 (± 6.6) Healthcare	Long-term nursing home residents	IG: individual & group health education	CG: usual care	8	Health status, self-efficacy	SR-PA	IG vs CG: ↑PA, ↑health status, ↑self-efficacy
[79]	Piedra LM – USA	2018	*N* = 572 (77%)	73.1 (± 6.8) Community	Community-dwelling older adults	IG: exercise program & group discussion	CG: health education & exercise program	104	Outcome expectations, QoL, self-efficacy	DM-PA; SR-PA	IG vs CG: ↑DB-PA, = SR-PA, = expectations, = QoL, = self-efficacy
[80]	Rausch O. AK-Switzerland	2021	*N* = 42 (50%)	67.9 (± 7.9) Healthcare	Outpatients with COPD	IG: Nordic Walking & COPD rehabilitation	CG: usual care	12	Exercise capacity, QoL, motivation	DM-PA; SR-PA, SR-SB	IG vs CG: = PA, = exercise capacity, = QoL, = motivation
[81]	Shimada H-Japan	2018	*N* = 308 (50%)	71.6 (± 5) Healthcare	Outpatients with mild cognitive impairment	IG: multicomponent exercise & pedometer	CG: health promotion classes & phone calls	40	Cognition	DM-PA	IG vs CG: ↑PA, ↑cognition
[82]	Siltanen S-Finland	2020	*N* = 204 (61%)	74–80 years Community	Community-dwelling older adults	IG: out-of-home activity counselling	CG: general health information	12	FF, outdoor & life-space mobility	DM-PA; SR-PA	IG vs CG: = PA, ↑FF, ↑outdoor mobility, = life-space mobility
[83]	Suikkanen S-Finland	2021	*N* = 299 (75%)	82.3 (± 6.3) Community	Community-dwelling older adults	IG: supervised exercise program	CG: usual daily activities	12	ADL, FF, handgrip strength, motor-cognition	SR-PA	IG vs CG: = PA, = ADL, ↑FF, = handgrip, = motor-cognition,
[84]	Thompson WG-USA	2014	*N* = 49 (80%)	79.5 (± 7) Community	Community-dwelling older adults	IG: accelerometer & feedback, counselling	CG: accelerometer without feedback	24	Anthropometry, FF	DM-PA	IG vs CG: = PA, = FF, = anthropometry (weight, % body fat)
[85]	Thomson RL-Australia	2014	*N* = 39 (56)	67.5 (± 6.6) Community	Community-dwelling older adults	IG: lutein capsules & PA promotion	CG: placebo & PA promotion	5	Weight, lutein, self-efficacy	DM-PA; DM-SB	IG vs CG: = PA, = SB, = weight, ↑plasma lutein, = self-efficacy
[86]	VanderW. NA-USA	2021	*N* = 55 (100%)	range: 65–84 (NA) Healthcare	Outpatients with breast cancer	IG: exercise book with walking diary	CG: usual care	4	Fatigue, FF, QoL, social support	SR-PA	IG vs CG: = PA, = fatigue, = FF, = QoL, = social support
[87]	Van Hoecke AS-Belgium	2014	*N* = 442 (66%)	69.5 (± 6.7) Community	Community-dwelling older adults	IG: PA individually tailored coaching	CG1: self-help booklet CG2: walking program	10	Motivation	DM-PA; SR-PA	IG vs CG1: ↑SR-PA, = motivation IG vs CG2: = PA, = motivation
[88]	Venditti EM-USA	2021	*N* = 322 (77%)	71.2 (± 4.3) Community	Community-dwelling older adults	IG: maintenance of PA & diet by phone, email	CG: PA newsletter maintenance	52	BMI, BP, FF	SR-PA	IG vs CG: = PA, ↓BMI (at 12 months), = BMI (at 24 months), = BP, = FF
[89]	Voukelatos A-Australia	2015	*N* = 385 (74%)	73.2 (NA) Community	Community-dwelling older adults	IG: walking program & email, phone coaching	CG: health-related emails & phone calls	26	Falls, fall efficacy, FF, mobility, QoL	SR-PA	IG vs CG: ↑PA, = falls, = fall efficacy, = QoL, ↑FF
[90]	Wan ES-USA	2017	*N* = 114 (2%)	68.6 (± 8.3) Healthcare	Outpatients with COPD	IG: pedometer & website with forum	CG: pedometer only	13	QoL, self-efficacy, social support, FF, motivation, COPD knowledge	DM-PA	IG vs CG: ↑PA, = QoL, = self-efficacy, = social support, = FF, = motivation, = COPD knowledge
[91]	Wang C-USA	2019	*N* = 44 (71%)	74.7 (± 6.4) Community	Community-dwelling older adults	IG: walking shoes & ankle–foot orthoses	CG: walking shoes only	26	Balance, FoF	DM-PA	IG vs CG: = PA, ↑balance, = FoF
[92]	Wang X-USA	2020	*N* = 61 (100)	65.5 (± 4.3) Community	Community-dwelling older adults	IG: MIPA walking, moderate dose	CG: MIPA walking, small dose	16	Cardiorespiratory fitness, weight	DM-PA; DM-SB	IG vs CG: = PA (↑daily steps), = SB, = cardiorespiratory fitness, = weight

*ADL* activities of daily living, *BMI* body mass index, *BP* blood pressure, *CG c*omparison group, *COPD* chronic obstructive pulmonary disease, *DM* device-measured, *FF* functional fitness, *FoF* fear of falling, *HIPA* high-intensity physical activity, *IG* intervention Group, *LIPA* low-intensity physical activity, *MIPA* moderate-intensity physical activity, *MVPA* moderate-to-vigorous intensity physical activity, *N* number, *NA* not available, *PA* physical activity, *PAD* peripheral artery disease, *QoL* quality of life, *SB* sedentary behaviours, *SR* self-reported, *SS* sample size, *TIA* transient ischemic attack

[N] Number according to the reference list; # publication year; * mean (standard deviation). Outcomes: =, ↑ and ↓ indicate no statistically significant differences, and statistically significant (*p* < 0.05) increases and decreases, respectively; “vs” indicates between-group interactions. § setting: Community or Healthcare

### Settings

The RCTs included in this study were conducted in a community setting (*n* = 30 [42–46, 48, 51, 52, 54, 56, 57, 61–64, 67, 68, 70, 76, 77, 79, 82–85, 87–89, 91, 92]), or a healthcare setting (*n* = 22 [41, 47, 49, 50, 53, 55, 58–60, 65, 66, 69, 71–75, 78, 80, 81, 86, 90]).

### Risk of bias

**Fig. 3:**
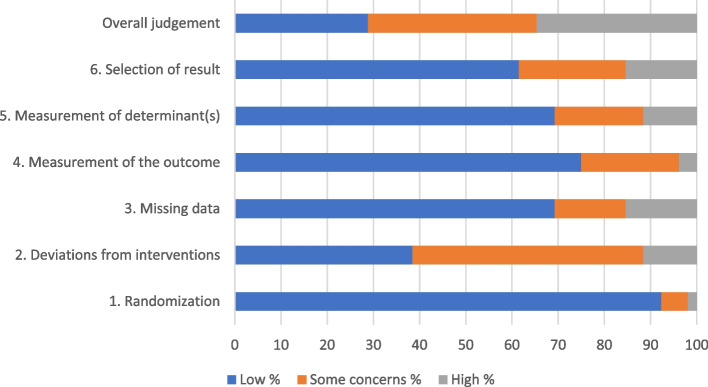
Risk of bias summary across all studies included in the review (*N* = 52)

The overall evaluation of the risk of bias is summarized in Fig. 3, whereas study-specific risk of bias results (*n* = 52) is available in Supplementary File 6.

A low risk of bias arising from the randomization process was identified in 48 (92%) of the studies. Conversely, the deviation from the intended interventions introduced bias in 26 (50%) of the studies (*n* = 24 and *n* = 2 using “intention-to-treat” and “per-protocol” approaches, respectively). A low risk of bias for 36 (69%) of the studies emerged for missing outcome data. In general, there was a low risk of bias for measurements of PA and SB outcomes (*n* = 39 studies; 75%) and determinants (*n* = 36 studies; 69%), whereas only 8 studies (15%) had a high risk of selective reporting. Overall, 19 studies (37%), 18 studies (35%), and 15 studies (29%) were judged to present some concerns, high risk, and low risk of bias, respectively.

### PA and SB outcomes

Twenty-three RCTs utilised exclusively device-measured PA and/or SB as outcomes [41, 49, 50, 53, 55, 56, 60, 61, 64–66, 71, 72, 74–77, 81, 84, 85, 90–92], while 17 RCTs relied solely on self-reported measures [42, 45–48, 51, 57, 58, 62, 63, 67, 73, 78, 83, 86, 88, 89]. Additionally, 12 RCTs utilised both device-based and self-report assessment methods [43, 44, 52, 54, 59, 68–70, 79, 80, 82, 87]. None of the studies focused exclusively on SB.

### Effects of interventions targeting modifiable determinants on device-measured PA

**Fig. 4:**
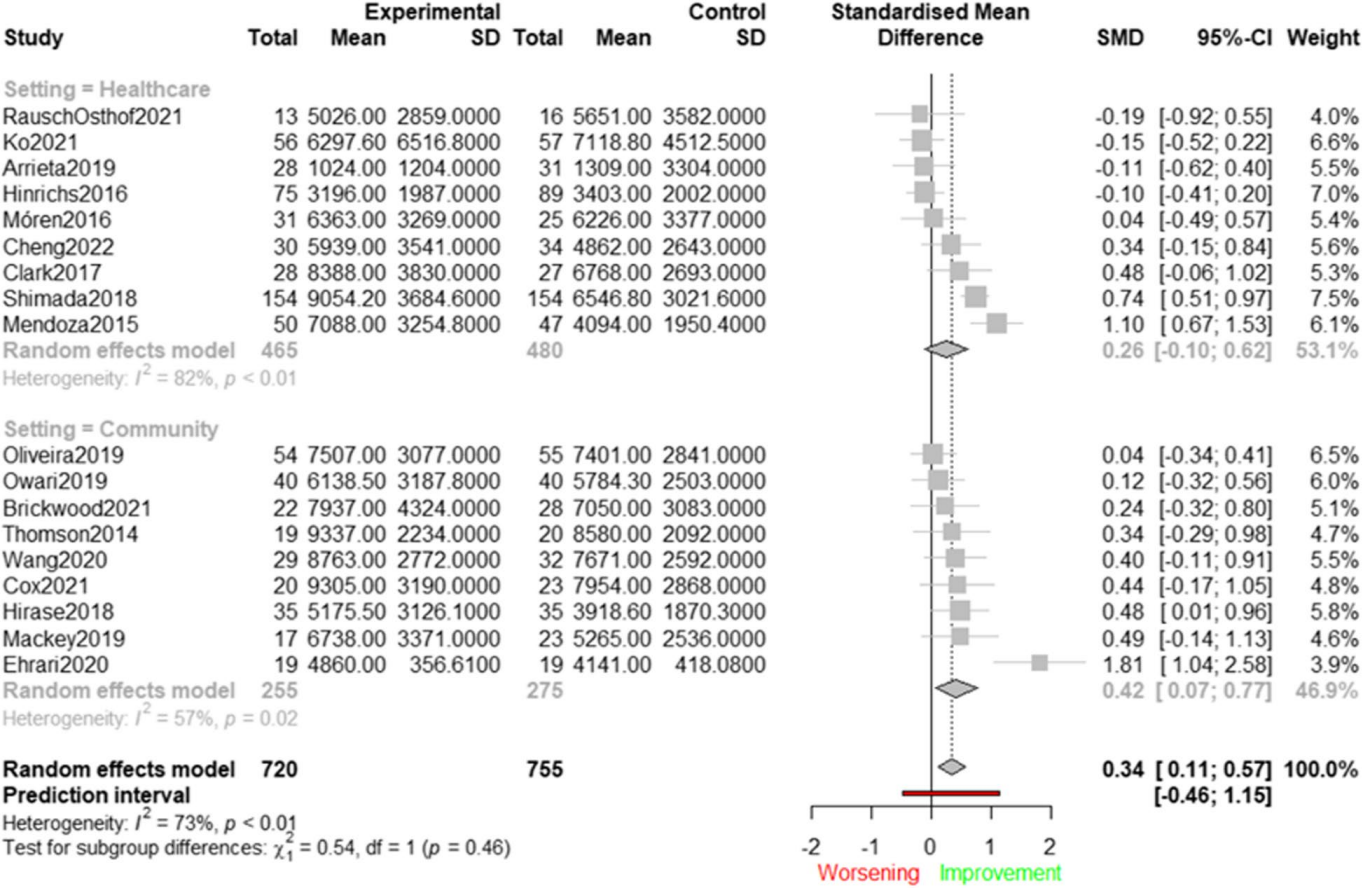
Effects of interventions targeting physical health and wellbeing determinants (steps/day)

Figure 4 shows the effects of 18 interventions aimed at enhancing device-measured PA (i.e., steps/day) by targeting physical health and wellbeing determinants [41, 44, 49, 50, 52, 56, 60, 61, 66, 68, 71, 72, 76, 77, 80, 81, 85, 92]. The average effect size across all studies was significant (SMD = 0.34; 95%CI: 0.11 to 0.57), indicating that interventions focusing on physical health and wellbeing determinants have the potential to increase device-measured steps in older adults. When re-expressing this SMD in terms of steps per day this finding corresponds to an average increase of 1,098 steps/day.

Statistically significant heterogeneity was observed (Q(17) = 63.27; *p* < 0.0001; I^2^ = 73.10%). Although setting did not emerge as a statistically significant moderator (Q = 0.54; *p* = 0.46), subgroup analysis revealed statistically significant effects for interventions conducted in a community setting (SMD = 0.42; 95%CI: 0.07 to 0.77), whereas non- statistically significant effects were found for those in a healthcare setting (SMD = 0.26; 95%CI: −0.10 to 0.62). Sensitivity analyses showed that the effect sizes largely remained within the 95% CI after removing potential outliers (SMD = 0.28; 95%CI: 0.10 to 0.47), and after removing low-quality studies (SMD = 0.34; 95%CI: 0.06 to 0.61) (see Supplementary File 3). Visual inspection of the funnel plot and results of the Egger’s Test (t[16] = −0.08; *p* = 0.83) suggested that publication bias was unlikely to have influenced the results (see Supplementary File 4).

**Fig. 5:**
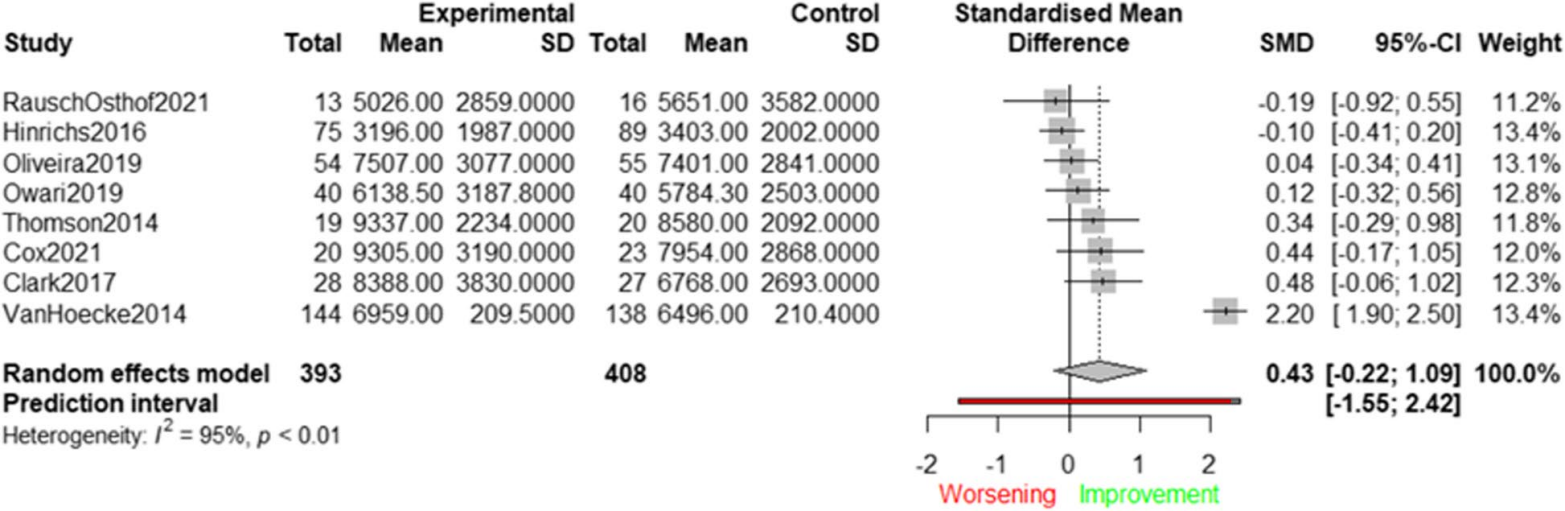
Effects of interventions targeting psychological and behavioural determinants (steps/day)

Figure 5 presents the results of eight studies targeting psychological and behavioural determinants on device-measured PA, expressed as steps/day, using a forest plot [50, 52, 60, 76, 77, 80, 85, 87].

The average effect size was not significant (SMD = 0.43; 95%CI: −0.22 to 1.09), indicating that existing interventions targeting psychological and behavioural determinants were not able to increase the numbers of steps/day. Significant heterogeneity was found (Q(7) = 150.48; *p* < 0.001; I^2^ = 95.3%), which was resolved after removing the one outlier study [87]. Sensitivity analyses were not performed due to the low number of studies. Visual inspection of the funnel plot suggested that publication bias was unlikely to have influenced the results (see Supplementary File 4). The Egger’s Test could not be administered due to the small number of studies. Forest plots displaying the results of the meta-analysis with MVPA revealed similar results (see Supplementary File 5). Briefly, studies targeting physical health and wellbeing determinants were able to increase MVPA (SMD = 0.41; 95%CI: 0.19 to 0.64). When re-expressing this SMD in terms of MVPA (min/day), we found an average increase of 12 min of MVPA/day. Subgroup analyses showed that significant effects were only found for studies conducted in the community setting (SMD = 0.53; 95%CI: 0.15 to 0.91). No significant effects were found for studies focusing on psychological and behavioural determinants (SMD = 0.26; 95%CI: −0.24 to 0.75).

### Effects of interventions targeting modifiable determinants on device-measured SB

**Fig. 6:**
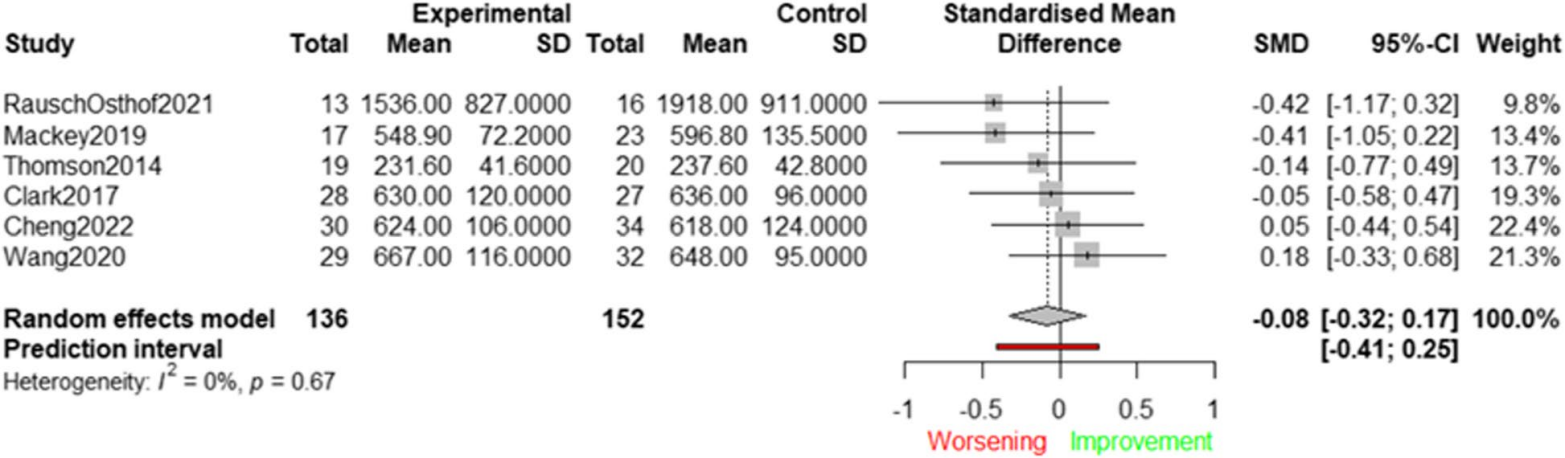
Effects of interventions targeting physical health and wellbeing determinants on device-measured SB (minutes/day)

Six of the included studies examined the effect of an intervention targeting physical health and wellbeing determinants on device-measured SB. Figure 6 presents the effects of these studies, as well as the average effect size by means of a forest plot.

The average effect size was not statistically significant (SMD = −0.08; 95%CI:—0.32 to 0.17), meaning that the existing interventions targeting physical health and wellbeing determinants were not able to reduce device-measured SB. No significant heterogeneity was found (Q(6) = 3.22; *p* = 0.67; I^2^ = 0.00%), and sensitivity analyses were not performed due to the low number of studies. Visual inspection of the funnel plot indicated that publication bias was unlikely to have influenced the results (see Supplementary File 4).

**Fig. 7:**
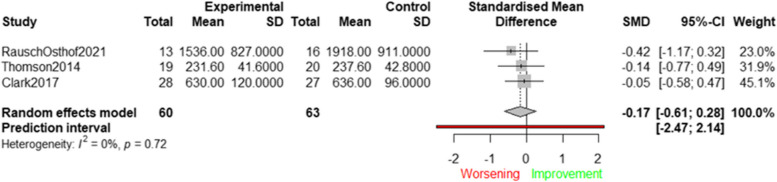
Effects of interventions targeting psychological and behavioural determinants on device-measured SB (minutes/day)

Only three studies investigated the effect of interventions targeting psychological and behaviour determinants on device-measured SB. Figure 7 displays the results of these studies, along with the average effect size represented in a forest plot.

The average effect size was not statistically significant (SMD = −0.17; 95%CI: −0.61 to 0.28), indicating that existing interventions targeting psychological and behaviour determinants did not lead to a reduction in device-measured SB. There was no significant heterogeneity detected among the studies (Q(3) = 0.65; *p* = 0.72; I^2^ = 0.00%), and sensitivity analyses were not conducted due to the limited number of studies. The funnel plot inspection suggested minimal likelihood of publication bias influencing the results (see Supplementary File 4).

## Discussion

The present SRMA summarized existing evidence regarding modifiable determinants of PA and SB among older adults, with a particular focus on the settings in which the intervention took place.

The first objective of this study was to identify modifiable determinants that have been targeted in intervention studies with PA and/or SB outcomes. Most of the studies focused on physical health and wellbeing determinants, such as physical functioning and quality of life. This finding is in line with the results of a scoping review of PA interventions in older adults suggesting that interventions were predominantly structured exercise programs, including balance and resistance training, and physical recreation, such as yoga and tai chi [93]. Physical health and wellbeing are critical for older adults’ PA and SB [94–96], yet the focus on them may have overshadowed other potentially impactful modifiable factors, such as psychological, behavioural, social, and environmental determinants. The underrepresentation of these other determinants in RCTs intervention studies suggests a missed opportunity to adopt a holistic, system-based approach to promote PA and reduce SB [97]. In fact, a system-based approach could consider how different determinants interact synergistically to influence behaviour, rather than focusing solely on biological parameters of physical functioning, although more easily measurable [97, 98]. Therefore, to develop more comprehensive intervention strategies further research is needed to explore and integrate underrepresented determinants in RCTs.

The second objective of the present study was to assess which of these interventions effectively increased PA and/or reduced SB in older adults. The meta-analyses revealed mixed results, with interventions targeting physical health and wellbeing determinants showing a moderate and statistically significant impact on increasing daily steps and MVPA, but not in reducing sedentary time. In contrast, interventions targeting psychological and behavioural determinants did not show significant effects on increasing PA or reducing SB behaviours, highlighting the need further refinement of interventions targeting psychological and behavioural determinants in older adults. It should also be noted that a significant number of studies were excluded because lacking information on determinants, whereas none of the included interventions had a primary focus on SB. Therefore, it is possible to speculate that this paucity of studies might have determined lack of evidence on modifiable SB determinants as an outcome. Additionally, the negative result from interventions targeting physical health and well-being determinants on SB were mainly derived from two studies only. In the first study, patients with chronic obstructive pulmonary disease were included, but according to the authors, the study suffered from low response rate leading to loss of statistical power [80]. In the second study [68] including community-dwelling older men, the intervention targeted PA and active transportation without specific focus on reducing sedentary time. The study by Rausch et al.[80] was also included in the meta-analyses as one of the three studies on determinants targeting psychological and behaviour determinants of SB. Despite that the use of device-measured data makes it possible to measure a range of variables, the majority of studies on PA and SB do not use an evaluation framework [99]. Thus, the variability in reporting and the inclusion of numerous outcome measures complicate the interpretation and comparison of results across different studies.

The third objective of this work was to assess if setting (community versus healthcare) influences the association between modifiable determinants and PA and SB.

Most of the interventions were delivered in a community setting, with interventions focusing on physical health and wellbeing determinants resulting particularly effective in community settings, where a high accessibility in neighbourhood environments, social interaction, and social support could increase the participants’ motivation to active lifestyles. However, more research is needed to explore whether and why community-based interventions may be more effective because this finding is inconsistent with previous research suggesting that delivery setting has no impact on the effectiveness of PA/SB interventions [100]. Clinical trials in older adults face challenges such as ageism, recruiting high-risk participants, managing multiple comorbidities and polypharmacy, ensuring protocol adherence, intervention compliance, safety, adverse event reporting, and standardizing geriatric-specific outcomes [101]. The RCT design in health care setting and evidence-based medicine has been criticized for the limited applicability for older adults as they are often excluded from clinical trials. In considering that a systematic review of 1,369 studies on RCTs in healthcare settings revealed that only 7% were specifically designed for older adults [102], thus, there is an urgent need for stronger evidence for increasing PA and reducing SB in an aging population.

### Strengths and limitations

This is the first study summarizing and evaluating existing and evidence on modifiable determinants of older adults’ PA and SB in community and healthcare settings.

The emphasis on intervention settings adds valuable insights. In fact, the effectiveness of specific determinants may vary depending on the context, working in one environment but not in another one [103]. For example, while community-based interventions might benefit from enhanced social support and accessibility, interventions in healthcare settings might lack these advantages. This setting-specific focus is crucial for tailoring interventions to maximize their effectiveness across different contexts and can thus be seen as an important strength of this review. Another important finding from this review is the heterogeneity in PA measurement across studies. The lack of standardization in how PA and SB are measured complicates the comparison of results and the synthesis of evidence. This issue is highlighted in recent literature, which underscores the need for more consistent and reliable methods of measuring PA and SB to improve the validity of research findings. Standardized measures would not only facilitate better comparison across studies but also enhance the ability to draw generalizable conclusions that can inform practice and policy.

This study presents also several limitations. By restricting the review to CTs and RCTs, the highest quality of evidence was obtained at the cost of a narrow focus. This approach might have excluded studies that explored natural and built environmental determinants, or those investigating political and economic aspects, which are often not targeted in controlled trials because of feasibility reasons. Recent research has identified environmental factors, such as walkability, as major determinants of PA [104]. The absence of such studies in this review highlights a crucial gap in the literature that needs to be addressed to fully understand the multifaceted nature of PA and SB determinants. Additionally, although not the primary focus of the current study, the presence or absence of an implementation plan is suggested to influence the effectiveness of community-based physical activity interventions [105]. RCTs often have relatively short follow-up periods, limiting the ability to assess how changes in the lives of older and oldest adults impact the results. A recent observational study on determinants of PA engagement in older adults found that both concurrent health and longitudinal changes in physical health significantly predicted PA levels. Furthermore, concurrent low mood also predicted PA levels, especially in older women [106]. Therefore, it can be speculated that the findings from our SRMA, which only includes RCTs, may differ from those of a meta-analysis on modifiable determinants on PA/SB that incorporates observational data from prospective studies. Furthermore, the risk of bias assessment revealed varying methodological rigor. Most studies had a robust randomization process, ensuring strong internal validity. However, deviations from intended interventions raised bias concerns in half of the studies, and consequently affected protocol adherence. Low risk of bias for missing data and outcome measurement suggests reliable data handling. Selective reporting was less common, indicating transparency. Whilst many studies were methodologically sound, a significant portion still faced issues affecting the reliability of their conclusions.

Regarding the research methodology, a limitation of this review is the absence of formal assessment of inter-rater agreement between screeners at the title/abstract, and full-text screening stages, such as calculating total agreement or Kappa statistics. While a consensus procedure was applied to resolve discrepancies, future reviews should include these measures to enhance methodological rigor.

Moreover, a DEDIPAC framework on key determinants of physical activity behaviours exists [9], but it was decided to provide a unified categorization of the determinants based on the Systems of Sedentary behaviours-framework [27]. Thus, future research could consider integrating these two approaches to provide a more comprehensive categorization for modifiable determinants of PA and SB. Additionally, the lack of homogeneity among the studies precluded more rigorous subgroup analyses or quantitative evaluations of heterogeneity. A high level of heterogeneity is also a major barrier to inter-study comparison or to later scale-up efforts. Future intervention studies on modifiable determinants should conduct contextual analyses prior to implementing complex interventions including older adults.

Another limitation of this study is that we focused solely on posttest differences to reflect the effects of the interventions, without incorporating baseline measurements or analyzing interaction effects (time × group). This decision was driven by the inconsistent reporting or absence of pretest data across studies, which made it challenging to include baseline measures in the analysis. While we assume minimal pretest differences between groups due to randomization in the included RCTs, the lack of baseline data limits our ability to confirm this assumption or to fully account for potential baseline imbalances. This could introduce some uncertainty into the interpretation of the pooled effect sizes. Future studies should strive for consistent reporting of pretest data to enable a more comprehensive analysis of intervention effects over time.

Lastly, an important limitation of our SRMA was the inability to consistently determine whether certain factors, for example quality of life and body mass index, were treated as determinants or outcome variables in the included studies. In many cases, this distinction was not clearly defined in the papers, making the interpretation of the results challenging. This uncertainty limited our ability to confidently ascertain whether these factors played a causal role in influencing PA and SB, or if they were themselves influenced by these behaviours. This ambiguity could have introduced potential biases in the analysis and highlights the need for future studies to more explicitly define the role of such variables. One could also question whether determinants of PA and SB can be accurately assessed from studies where exercise or physical activity is the intervention. However, we posit that physical functioning and physical fitness, as evaluated in intervention studies where PA/SB is the primary focus, can indeed be considered determinants of PA. This is because physical functioning and physical fitness, encompassing attributes such as strength, endurance, oxygen uptake, and flexibility, significantly influence an older adult's capacity to engage in and maintain physical activity. These physical determinants can only be effectively addressed through the implementation of exercise, resistance, and/or strength interventions. Conversely, if the primary objective is to ascertain whether baseline physical function or fitness levels in older adults can predict future PA/SB levels, this would be more appropriately examined through meta-analyses of prospective population-based studies.

### Potential for policy development and complex interventions

Despite the limitations, the findings from this SRMA offer promising insights for the development of policies and complex interventions aimed at increasing PA and reducing SB. By focusing on the physical health and wellbeing determinants identified in the review, policymakers can design targeted actions that address the root causes of PA/SB in older adults. This is particularly important given the aging population and the growing public health challenge posed by high levels of inactivity. While community-based interventions could take advantage of targeted policies prioritizing accessibility and social engagement, programs in healthcare settings must integrate tailored strategies into routine care, fostering interdisciplinary approaches for promoting active and healthy aging. However, our findings also highlight key gaps in knowledge, particularly in understanding how less frequently studied determinants, such as psychological and social factors, contribute to PA and SB.

## Conclusion

This SRMA suggests that physical health and wellbeing determinants could be relevant targets to increase PA in older adults, particularly in community settings. However, the underrepresentation of psychological, behavioural, social, and cultural determinants suggests a need for a more holistic approach. Future research should explore these underrepresented factors to develop comprehensive intervention strategies. Additionally, understanding the varying effectiveness of interventions across different settings remains crucial for optimizing outcomes in an aging population.

## Supplementary Information

Supplementary Material 1

Supplementary Material 2

Supplementary Material 3

Supplementary Material 4

Supplementary Material 5

Supplementary Material 6

## Data Availability

The datasets generated and/or analyzed during the current study are available from the corresponding author upon reasonable request.

## Appendix

### List of acronyms

APA: American Psychology Association

COST: Cooperation in the Field of Science and Technology Research (Europe)

COVID-19: Coronavirus Disease 2019

CT: Control Trial

DEDIPAC: Determinants of Diet and Physical Activity Knowledge

DE-PASS: Determinants of Physical Activities in Settings

EBSCO: Elton B. Stephens CO (company)

JASP: Jeffreys's Amazing Statistics Program

MA: Meta Analysis

MCMC: Markov Chain Monte Carlo (algorithm)

Meta-SEM: Meta-Analytical Structural Equation Modelling

METs: Metabolic Equivalent of Tasks

PA: Physical Activity

PRISMA: Preferred Reporting Items for Systematic Reviews and Meta-Analyses

PROSPERO: International Prospective Register of Systematic Reviews

RCT: Randomised Control Trial

RoB 2.0: Revised Cochrane risk of bias tool for randomized trials

ROBINS-I: Risk Of Bias In Non-randomized Studies - of Interventions

RobMA: Robust Bayesian Meta-Analysis

SB: Sedentary Behaviours

SLR: Systematic Literature Review

SMD: Standardized mean difference

SRMA: Systematic Review and Meta-analysis

WHO: World Health Organization
