# Supplementary material for: Modifiable determinants of older adults’ physical activity and sedentary behavior in community and healthcare settings: a DE-PASS systematic review and meta-analysis
Source: Eur Rev Aging Phys Act. 2025 May 24;22:9. doi: 10.1186/s11556-025-00373-y (PMC12103017; doi:10.1186/s11556-025-00373-y)
Supplement: Supplementary file 1 — Supplementary Material 1 [file 11556_2025_373_MOESM1_ESM.docx]

**Supplementary File 1.** Full search strategy for the selected databases.

**PubMed**:

Search: **("Physical activ*") OR (exercis*) OR (sport*) OR (play*) OR (exertion*) OR (recreation) OR (training) OR ("motor activit*") OR ("physical performance") OR ("physical mov*") OR ("physical effort") OR (exergam*) OR (workout) OR (sedentar*) OR ("screen time") OR (gaming) OR ("computer use") OR ("using computer") OR (sitting) OR (inactiv*) OR ("seated postur*") OR ("watch television") OR ("watch TV") OR ("view television") OR ("view TV") OR (relax*) OR (rest*) AND (RCT) OR ("control* trial*") OR (quasi) OR (intervention*) AND (determinant*) OR (antecedent*) OR (predictor*) OR (mediator*) OR (moderator*) OR (exposure*) AND (old*) OR (elder*) OR (adult*) OR ("aged people") OR ("ageing*") OR (senior*) OR (veteran*) OR (mature*) AND (survey*) OR (questionnaire*) OR ("activity profile") OR (recall) OR (diary) OR ("activity monitor*") OR ("heart rate monitor*") OR ("direct observation*") OR (actigraph*) OR ("activity track*") OR ("self report*") OR (pedomet*) OR (wearable*) OR (acceleromet*)** Filters: **Clinical Trial, Controlled Clinical Trial, Randomized Controlled Trial, in the last 10 years, Humans, Female, Male, Aged: 65+ years, 80 and over: 80+ years** Sort by: **Publication Date**

((((((((("physical activ*"[All Fields] OR "exercis*"[All Fields] OR "sport*"[All Fields] OR "play*"[All Fields] OR "exertion*"[All Fields] OR ("recreation"[MeSH Terms] OR "recreation"[All Fields] OR "recreations"[All Fields] OR "recreational"[All Fields] OR "recreator"[All Fields] OR "recreators"[All Fields]) OR ("education"[MeSH Subheading] OR "education"[All Fields] OR "training"[All Fields] OR "education"[MeSH Terms] OR "train"[All Fields] OR "train s"[All Fields] OR "trained"[All Fields] OR "training s"[All Fields] OR "trainings"[All Fields] OR "trains"[All Fields]) OR "motor activit*"[All Fields] OR "physical performance"[All Fields] OR "physical mov*"[All Fields] OR "physical effort"[All Fields] OR "exergam*"[All Fields] OR ("workout"[All Fields] OR "workouts"[All Fields]) OR "sedentar*"[All Fields] OR "screen time"[All Fields] OR ("game s"[All Fields] OR "games"[All Fields] OR "gaming"[All Fields]) OR "computer use"[All Fields] OR "using computer"[All Fields] OR ("sitting position"[MeSH Terms] OR ("sitting"[All Fields] AND "position"[All Fields]) OR "sitting position"[All Fields] OR "sitting"[All Fields] OR "sittings"[All Fields]) OR "inactiv*"[All Fields] OR "seated postur*"[All Fields] OR "watch television"[All Fields] OR "watch TV"[All Fields] OR "view television"[All Fields] OR (("view beijing"[Journal] OR "view"[All Fields]) AND "tv"[All Fields]) OR "relax*"[All Fields] OR "rest*"[All Fields]) AND "RCT"[All Fields]) OR "control trial*"[All Fields] OR "quasi"[All Fields] OR "intervention*"[All Fields]) AND "determinant*"[All Fields]) OR "antecedent*"[All Fields] OR "predictor*"[All Fields] OR "mediator*"[All Fields] OR "moderator*"[All Fields] OR "exposure*"[All Fields]) AND "old"[All Fields]) OR "elder*"[All Fields] OR "adult*"[All Fields] OR "aged people"[All Fields] OR "ageing*"[All Fields] OR "senior*"[All Fields] OR "veteran*"[All Fields] OR "mature*"[All Fields]) AND "survey*"[All Fields]) OR "questionnaire*"[All Fields] OR "activity profile"[All Fields] OR ("mental recall"[MeSH Terms] OR ("mental"[All Fields] AND "recall"[All Fields]) OR "mental recall"[All Fields] OR "recall"[All Fields] OR "recalling"[All Fields] OR "recallable"[All Fields] OR "recalled"[All Fields] OR "recallers"[All Fields] OR "recalls"[All Fields]) OR ("diaries"[All Fields] OR "diary"[All Fields]) OR "activity monitor*"[All Fields] OR "heart rate monitor*"[All Fields] OR "direct observation*"[All Fields] OR "actigraph*"[All Fields] OR "activity track*"[All Fields] OR "self report*"[All Fields] OR "pedomet*"[All Fields] OR "wearable*"[All Fields] OR "acceleromet*"[All Fields]) AND ((y_10[Filter]) AND (clinicaltrial[Filter] OR controlledclinicaltrial[Filter] OR randomizedcontrolledtrial[Filter]) AND (humans[Filter]) AND (female[Filter] OR male[Filter]) AND (aged[Filter] OR 80andover[Filter]))

**Translations**

**recreation:** "recreation"[MeSH Terms] OR "recreation"[All Fields] OR "recreations"[All Fields] OR "recreational"[All Fields] OR "recreator"[All Fields] OR "recreators"[All Fields]

**training:** "education"[Subheading] OR "education"[All Fields] OR "training"[All Fields] OR "education"[MeSH Terms] OR "train"[All Fields] OR "train's"[All Fields] OR "trained"[All Fields] OR "training's"[All Fields] OR "trainings"[All Fields] OR "trains"[All Fields]

**workout:** "workout"[All Fields] OR "workouts"[All Fields]

**gaming:** "game's"[All Fields] OR "games"[All Fields] OR "gaming"[All Fields]

**sitting:** "sitting position"[MeSH Terms] OR ("sitting"[All Fields] AND "position"[All Fields]) OR "sitting position"[All Fields] OR "sitting"[All Fields] OR "sittings"[All Fields]

**recall:** "mental recall"[MeSH Terms] OR ("mental"[All Fields] AND "recall"[All Fields]) OR "mental recall"[All Fields] OR "recall"[All Fields] OR "recalling"[All Fields] OR "recallable"[All Fields] OR "recalled"[All Fields] OR "recallers"[All Fields] OR "recalls"[All Fields]

**diary:** "diaries"[All Fields] OR "diary"[All Fields]

**Warnings**

("Physical activ*") OR (exercis*) OR (sport*) OR (play*) OR (exertion*) OR (recreation) OR (training) OR ("motor activit*") OR ("physical performance") OR ("physical mov*") OR ("physical effort") OR (exergam*) OR (workout) OR (sedentar*) OR ("screen time") OR (gaming) OR ("computer use") OR ("using computer") OR (sitting) OR (inactiv*) OR ("seated postur*") OR ("watch television") OR ("watch TV") OR ("view television") OR ("**view TV**") OR (relax*) OR (rest*) AND (RCT) OR ("control* trial*") OR (quasi) OR (intervention*) AND (determinant*) OR (antecedent*) OR (predictor*) OR (mediator*) OR (moderator*) OR (exposure*) AND (old*) OR (elder*) OR (adult*) OR ("aged people") OR ("ageing*") OR (senior*) OR (veteran*) OR (mature*) AND (survey*) OR (questionnaire*) OR ("activity profile") OR (recall) OR (diary) OR ("activity monitor*") OR ("heart rate monitor*") OR ("direct observation*") OR (actigraph*) OR ("activity track*") OR ("self report*") OR (pedomet*) OR (wearable*) OR (acceleromet*)

**Quoted phrase not found:** view TV

15,567 results

Filters applied: *Clinical Trial, Controlled Clinical Trial, Randomized Controlled Trial, in the last 10 years, Humans, Female, Male, Aged: 65+ years, 80 and over: 80+ years*.

The asterisk in your search was ignored. You must use 4 or more characters for a wildcard search. Lengthen the root word to search for all endings.

Quoted phrase not found: "view TV"

**EBSCOhost**:

S1

( ("Physical activ*") OR (exercis*) OR (sport*) OR (play*) OR (exertion*) OR (recreation) OR (training) OR ("motor activit*") OR ("physical performance") OR ("physical mov*") OR ("physical effort") OR (exergam*) OR (workout) ) OR ( (sedentar*) OR ("screen time") OR (gaming) OR ("computer use") OR ("using computer") OR (sitting) OR (inactiv*) OR ("seated postur*") OR ("watch television") OR ("watch TV") OR ("view television") OR ("view TV") OR (relax*) OR (rest*) ) AND ( (RCT) OR ("control* trial*") OR (quasi) OR (intervention*) ) AND ( (determinant*) OR (antecedent*) OR (predictor*) OR (mediator*) OR (moderator*) OR (exposure*) ) AND ( (old*) OR (elder*) OR (adult*) OR ("aged people") OR ("ageing*") OR (senior*) OR (veteran*) OR (mature*) ) AND ( (survey*) OR (questionnaire*) OR ("activity profile") OR (recall) OR (diary) OR ("activity monitor*") OR ("heart rate monitor*") OR ("direct observation*") OR (actigraph*) OR ("activity track*") OR ("self report*") OR (pedomet*) OR (wearable*) OR (acceleromet*) ) Show Less

Limiters - Published Date: 20120101-20221231; Scholarly (Peer Reviewed) Journals; Document Type: Journal Article; Year of Publication: 2021-2022; Age Groups: Aged (65 yrs & older), Very Old (85 yrs & older); Population Group: Human; Methodology: CLINICAL TRIAL; Publication Type: Academic Journal

Expanders - Apply equivalent subjects

Search modes - Boolean/Phrase

[**Results**](javascript:__doPostBack('ctl00$ctl00$FindField$FindField$historyControl$HistoryRepeater$ctl02$linkResults',''))**(17)**

# Last Run

## Interface

 - EBSCOhost Research Databases

## Search Screen

 - Advanced Search

## Database

 - APA PsycArticles;SPORTDiscus with Full Text

**Web Of Science**:

**("Physical activ*") OR (exercis*) OR (sport*) OR (play*) OR (exertion*) OR (recreation) OR (training) OR ("motor activit*") OR ("physical performance") OR ("physical mov*") OR ("physical effort") OR (exergam*) OR (workout)** (All Fields) or **(sedentar*) OR ("screen time") OR (gaming) OR ("computer use") OR ("using computer") OR (sitting) OR (inactiv*) OR ("seated postur*") OR ("watch television") OR ("watch TV") OR ("view television") OR ("view TV") OR (relax*) OR (rest*)** (All Fields) and **(RCT) OR ("control* trial*") OR (quasi) OR (intervention*)** (All Fields) and **(determinant*) OR (antecedent*) OR (predictor*) OR (mediator*) OR (moderator*) OR (exposure*)** (All Fields) and **(old*) OR (elder*) OR (adult*) OR ("aged people") OR ("ageing*") OR (senior*) OR (veteran*) OR (mature*)** (All Fields) and **(survey*) OR (questionnaire*) OR ("activity profile") OR (recall) OR (diary) OR ("activity monitor*") OR ("heart rate monitor*") OR ("direct observation*") OR (actigraph*) OR ("activity track*") OR ("self report*") OR (pedomet*) OR (wearable*) OR (acceleromet*)** (All Fields) and **2022** or **2021** or **2020** or **2019** or **2018** or **2017** or **2016** or **2015** or **2014** or **2013** or **2012** (Publication Years) and **Articles** (Document Types) and **Articles** (Document Types) and **Multidisciplinary Sciences** or **Neurosciences** or **Environmental Sciences** or **Public Environmental Occupational Health** or **Sport Sciences** or **Medicine General Internal** or **Education Educational Research** or **Medicine Research Experimental** or **Physiology** or **Rehabilitation** or **Cardiac Cardiovascular Systems** or **Health Care Sciences Services** or **Hospitality Leisure Sport Tourism** or **Psychology Multidisciplinary** or **Environmental Studies** or **Gerontology** or **Psychology Applied** or **Psychology Experimental** or **Behavioral Sciences** or **Social Sciences Interdisciplinary** or **Psychology** or **Psychology Social** or **Psychology Educational** or **Neuroimaging** or **Ergonomics** (Web of Science Categories) and **Engineering** or **Cardiovascular System Cardiology** or **Pharmacology Pharmacy** or **Surgery** or **Business Economics** or **Cell Biology** or **Water Resources** or **Biochemistry Molecular Biology** or **Oncology** or **Geology** or **Meteorology Atmospheric Sciences** or **Biotechnology Applied Microbiology** or **Computer Science** or **Radiology Nuclear Medicine Medical Imaging** or **Medical Informatics** or **Remote Sensing** or **Zoology** or **Linguistics** or **Imaging Science Photographic Technology** or **Immunology** or **Toxicology** or **Biomedical Social Sciences** or **Respiratory System** or **Marine Freshwater Biology** or **Biodiversity Conservation** or **Anatomy Morphology** or **Entomology** or **Religion** or **Arts Humanities Other Topics** or **History Philosophy Of Science** or **Nuclear Science Technology** or **Microbiology** or **Gastroenterology Hepatology** or **Forestry** or **Developmental Biology** or **Transplantation** or **Anthropology** or **Urology Nephrology** or **Information Science Library Science** or **Obstetrics Gynecology** or **Social Issues** or **Veterinary Sciences** or **Government Law** or **Communication** or **Music** or **Otorhinolaryngology** or **Criminology Penology** or **Plant Sciences** or **Women S Studies** or **Family Studies** or **History** or **Physical Geography** or **Anesthesiology** or **Tropical Medicine** or **Chemistry** or **Social Work** or **Substance Abuse** or **Hematology** or **Biophysics** or **Energy Fuels** or **Pathology** or **Mathematical Computational Biology** or **Nursing** or **Geography** or **Agriculture** or **Transportation** or **Public Administration** or **Infectious Diseases** or **Mathematics** or **Audiology Speech Language Pathology** or **Urban Studies** or **Pediatrics** or **International Relations** or **Ophthalmology** or **Philosophy** or **Ethnic Studies** or **Development Studies** or **Robotics** or **Fisheries** or **Medical Laboratory Technology** or **Mathematical Methods In Social Sciences** or **Emergency Medicine** or **Food Science Technology** or **Dermatology** or **Demography** or **Dentistry Oral Surgery Medicine** or **Construction Building Technology** or **Virology** or **Cultural Studies** or **Area Studies** or **Operations Research Management Science** or **Art** or **Medical Ethics** or **Theater** or **Parasitology** or **Evolutionary Biology** or **Oceanography** or **Legal Medicine** or **Materials Science** or **Mechanics** or **Instruments Instrumentation** or **Film Radio Television** or **Optics** or **Architecture** or **Rheumatology** or **Astronomy Astrophysics** or **Archaeology** or **Physics** or **Literature** or **Mining Mineral Processing** or **Spectroscopy** or **Allergy** or **Geochemistry Geophysics** or **Mineralogy** or **Mycology** or **Reproductive Biology** or **Microscopy** (Exclude – Research Areas) and **42ND ANNUAL MEETING OF INTERNATIONAL SOCIETY ON OXYGEN TRANSPORT TO TISSUE** or **10TH ANNUAL MILITARY HEALTH SYSTEM RESEARCH SYMPOSIUM MHSRS** or **MILITARY HEALTH SYSTEM RESEARCH SYMPOSIUM MHSRS** or **WINTER CONFERENCES OF SPORTS SCIENCE COSTA BLANCA SPORTS SCIENCE EVENTS** or **CONFERENCE ON NEUROSCIENCES AND MUSIC IV LEARNING AND MEMORY** or **MILITARY HEALTH SCIENCES SYSTEM RESEARCH SYMPOSIUM MHSRS** or **45TH ANNUAL MEETING OF THE INTERNATIONAL SOCIETY ON OXYGEN TRANSPORT TO TISSUE ISOTT** or **43RD ANNUAL MEETING OF THE INTERNATIONAL SOCIETY ON OXYGEN TRANSPORT TO TISSUE ISOTT** or **46TH ANNUAL MEETING OF THE INTERNATIONAL SOCIETY ON OXYGEN TRANSPORT TO TISSUE ISOTT** or **6TH INTERNATIONAL CONFERENCE ON NEUROSCIENCES AND MUSIC** or **44TH ANNUAL MEETING OF THE INTERNATIONAL SOCIETY ON OXYGEN TRANSPORT TO TISSUE ISOTT** or **40TH ANNUAL MEETING OF THE INTERNATIONAL SOCIETY ON OXYGEN TRANSPORT TO TISSUE ISOTT** or **24TH ANNUAL MEETING OF THE SOCIETY FOR THE STUDY OF INGESTIVE BEHAVIOR SSIB** or **42ND ANNUAL MEETING OF THE INTERNATIONAL SOCIETY ON OXYGEN TRANSPORT TO TISSUE ISOTT** or **INTERNATIONAL CONFERENCE ON COMMUNICATION IN HEALTHCARE ICCH** or **2014 GLOBAL SUMMIT ON THE PHYSICAL ACTIVITY OF CHILDREN** or **4TH WORLD CONGRESS ON GENETICS GERIATRICS AND NEURODEGENERATIVE DISEASES RESEARCH GENEDIS** or **CONFERENCE ON NEUROSCIENCES AND MUSIC V** or **INTERIM MEETING OF THE AMERICAN ORTHOPAEDIC SOCIETY FOR SPORTS MEDICINE AOSSM** or **22ND INTERNATIONAL CONGRESS ON SNOW SPORTS TRAUMA AND SAFETY BIENNIAL MEETING OF THE INTERNATIONAL SOCIETY FOR SKIING SAFETY ISSS** or **25TH ANNUAL MEETING OF THE SOCIETY FOR THE STUDY OF INGESTIVE BEHAVIOR SSIB** or **37TH ANNUAL MEETING OF THE AMERICAN ORTHOPAEDIC SOCIETY FOR SPORTS MEDICINE AOSSM** or **INTERNATIONAL MULTIDISCIPLINARY SEMINAR STRESA HEADACHE** or **2ND WORLD CONGRESS ON GENETICS GERIATRICS AND NEURODEGENERATIVE DISEASE RESEARCH GENEDIS** or **6TH INTERNATIONAL CONFERENCE ON SODIUM CALCIUM EXCHANGE** or **19TH INTERNATIONAL MESAEP SYMPOSIUM ON ENVIRONMENTAL POLLUTION AND ITS IMPACT ON LIFE IN THE MEDITERRANEAN REGION** or **8TH NORDIC HEALTH PROMOTION RESEARCH CONFERENCES NHPRC ON 20 YEARS OF HEALTH PROMOTION RESEARCH IN THE NORDIC COUNTRIES HEALTH WELLBEING AND PHYSICAL ACTIVITY** or **MILITARY HEALTH SYSTEM RESEARCH SYMPOSIUM** or **11TH INTERNATIONAL SYMPOSIUM ON RECENT ADVANCES IN ENVIRONMENTAL HEALTH RESEARCH** or **MEETING OF THE OLYMPIC STUDIES FORUM** or **10TH CONFERENCE OF THE INTERNATIONAL SHOULDER GROUP** or **2ND INTERNATIONAL CONFERENCE ON PHYSICAL EMPLOYMENT STANDARDS PES** or **3RD INTERNATIONAL CONFERENCE ON PHYSICAL EMPLOYMENT STANDARDS PES** or **44TH ANNUAL MEETING OF THE AMERICAN ORTHOPAEDIC SOCIETY FOR SPORTS MEDICINE AOSSM** or **62ND ANNUAL MEETING OF THE AMERICAN COLLEGE OF SPORTS MEDICINE** or **CONTAMINATED LAND ECOLOGICAL ASSESSMENT AND REMEDIATION CONFERENCE SERIES CLEAR** or **INTERNATIONAL CONFERENCE ON EDUCATION RESEARCH ICER** or **INTERNATIONAL SCIENTIFIC AND PROFESSIONAL CONFERENCE ON PRESENT FOR THE FUTURE OF EDUCATION OPPORTUNITIES AND CHALLENGES** or **SUSTAINABLE ASIA CONFERENCE SAC** or **10TH INTERNATIONAL CONFERENCE ON ALZHEIMERS AND PARKINSONS DISEASES AD PD** or **18TH INTERNATIONAL SYMPOSIUM ON ENVIRONMENTAL POLLUTION AND ITS IMPACT ON LIFE IN THE MEDITERRANEAN REGION MESAEP** or **18TH MEETING OF THE INTERNATIONAL SOCIETY FOR ARTERIAL CHEMORECEPTION** or **22ND INTERNATIONAL SYMPOSIUM ON SHIFTWORK AND WORKING TIME** or **27TH ANNUAL MEETING OF THE SOCIETY FOR THE STUDY OF INGESTIVE BEHAVIOR SSIB** or **45TH ANNUAL MEETING OF THE AMERICAN ORTHOPAEDIC SOCIETY FOR SPORTS MEDICINE AOSSM** or **4TH INTERNATIONAL CONGRESS OF SOLDIERS PHYSICAL PERFORMANCE ICSPP** or **COMBINED SECTIONS MEETING OF THE AMERICAN PHYSICAL THERAPY ASSOCIATION** or **CONFERENCE OF THE EUROPEAN ASSOCIATION FOR COMMUNICATION IN HEALTHCARE EACH** or **CONFERENCE ON CELLULAR AND NETWORK FUNCTIONS IN THE SPINAL CORD** or **CONFERENCE ON INTEGRATED SCIENCE AND KNOWLEDGE CO PRODUCTION FOR ECOSYSTEMS AND HUMAN WELL BEING** or **INTERNATIONAL CONFERENCE ON CONTAMINATED SEDIMENTS CONTASED 2015** or **22ND ANNUAL MEETING OF THE SOCIETY FOR THE STUDY OF INGESTIVE BEHAVIOR** or **38TH ANNUAL CONFERENCE OF THE INTERNATIONAL SOCIETY ON OXYGEN TRANSPORT TO TISSUE ISOTT** or **43RD ANNUAL MEETING OF THE AMERICAN ORTHOPAEDIC SOCIETY FOR SPORTS MEDICINE AOSSM** or **4TH INTERNATIONAL CONFERENCE ON CONTAMINATED LAND ECOLOGICAL ASSESSMENT AND REMEDIATION CLEAR GREEN AND SUSTAINABLE LAND REMEDIATION** or **CONFERENCE ON RESEARCHING PARADIGMS OF CHILDHOOD AND EDUCATION** or **INTERNATIONAL WORKSHOP ON NEURAL CODING** or **11TH INTERNATIONAL CONFERENCE ON DEVELOPMENTAL COORDINATION DISORDER DCD** or **17TH MATE DEMARIN DAYS ON EDUCATION THE FUTURE OF CIVILIZATION** or **1ST INTERNATIONAL CONFERENCE ON PAST PRESENT AND FUTURE OF PUBLIC HEALTH IN EAST ASIA** or **23RD INTERNATIONAL SYMPOSIUM ON SHIFTWORK AND WORKING TIME TOWARDS A GLOBAL CONSENSUS** or **26TH ANNUAL MEETING OF THE INTERNATIONAL BEHAVIORAL NEUROSCIENCE SOCIETY IBNS CONTEMPORARY CONTRIBUTIONS TO BASIC AND TRANSLATIONAL BEHAVIORAL NEUROSCIENCE RESEARCH** or **2ND CONTAMINATED LAND ECOLOGICAL ASSESSMENT AND REMEDIATION CLEAR 2014 CONFERENCE ENVIRONMENTAL POLLUTION AND REMEDIATION** or **38TH WORLD CONGRESS OF THE INTERNATIONAL UNION OF PHYSIOLOGICAL SCIENCES** or **3RD PANGU STROKE CONFERENCE** or **3RD RESEARCH SYMPOSIUM OF THE AUSTRALASIAN JOURNAL OF EARLY CHILDHOOD AJEC** or **PAEDIATRIC VIROLOGY WORKSHOP** or **PROCEEDINGS OF THE SIXTH INTERNATIONAL CONFERENCE ON SPATIAL COGNITION ICSC** or **10TH ANNUAL INTERNATIONAL CONFERENCE ON CHALLENGES IN ENVIRONMENTAL SCIENCE AND ENGINEEERING CESE** or **11TH INTERNATIONAL CONFERENCE ON ALZHEIMER S AND PARKINSON S DISEASES AD PD** or **14TH MATE DEMARIN DAYS INTERNATIONAL SCIENTIFIC SYMPOSIUM** or **25TH TOPICAL MEETING OF THE INTERNATIONAL SOCIETY OF ELECTROCHEMISTRY ISE** or **2ND INTERNATIONAL CONFERENCE ON GLOBAL TELEHEALTH** or **38TH ANNUAL CONFERENCE OF THE INTERNATIONAL STANDING CONFERENCE FOR THE HISTORY OF EDUCATION ISCHE** or **8TH ANNUAL CONFERENCE INTERNATIONAL PHYTOTECHNOLOGY SOCIETY PUTTING PLANTS TO WORK WHERE WE LIVE LABOR BREATHE AND PLAY** or **8TH INTERNATIONAL SYMPOSIUM OF THE SOCIETY FOR RESEARCH ON THE CEREBELLUM AND ATAXIA SRCA** or **ANNUAL CONFERENCE OF THE AMERICAN ANTHROPOLOGICAL ASSOCIATION** or **ANNUAL SYMPOSIUM OF THE SOCIETY FOR THE STUDY OF INBORN ERRORS OF METABOLISM SSIEM** or **EXPERIMENTAL BIOLOGY MEETING 2015** or **MILITARY HEALTH SYSTEMS RESEARCH SYMPOSIUM MHSRS** or **SCIENTIFIC MEETING ON AUSTRALIA AND NEW ZEALAND DEVELOPMENTAL ORIGINS OF HEALTH AND DISEASE ANZ DOHAD** or **SYMPOSIUM ON BLUE VERSUS GRAY POTENTIAL HEALTH BENEFITS OF BLUEBERRIES FOR SUCCESSFUL AGING HELD AT THE INTERNATIONAL CONGRESS OF GERONTOLOGY** or **SYMPOSIUM ON THE ROLE OF RENAL NERVES INCARDIOVASCULAR AND RENAL FUNCTION IN NORMAL AND PATHOPHYSIOLOGICAL STATES** or **10TH INTERNATIONAL WORKSHOP ON NEURAL CODING NC** or **12TH FORUM OF THE INTERNATIONAL HCH AND PESTICIDES ASSOCIATION IHPA** or **12TH INTERNATIONAL SYMPOSIUM ON RECENT ADVANCES IN ENVIRONMENTAL HEALTH RESEARCH** or **17TH INTERNATIONAL CONFERENCE ON BRAIN EDEMA AND CELLULAR INJURY** or **25TH ANNUAL INJURY FREE COALITION FOR KIDS CONFERENCE FORGING NEW FRONTIERS CHANGING THE CONVERSATION ON GUN SAFETY** or **2ND INTERNATIONAL COMFORT CONGRESS ICC** or **2ND INTERNATIONAL CONFERENCE ON MATERNAL AND NEWBORN HEALTH TRANSLATING RESEARCH EVIDENCE TO PRACTICE** or **39TH ANNUAL MEETING OF THE AMERICAN ORTHOPAEDIC SOCIETY FOR SPORTS MEDICINE AOSSM** or **3RD EURASIAN CONFERENCE ON EDUCATIONAL INNOVATION ECEI** or **3RD INTERNATIONAL CONFERENCE ON MICROELECTRONICS COMPUTING AND COMMUNICATION SYSTEMS MCCS** or **56TH ANNUAL RESEARCH IN MEDICAL EDUCATION SESSIONS RIME OF THE ASSOCIATION OF AMERICAN MEDICAL COLLEGES AAMC** or **5TH GLOBAL SYMPOSIUM ON HEALTH SYSTEMS RESEARCH** or **5TH INTERNATIONAL CONFERENCE AUDITORY CORTEX** or **5TH MILITARY HEALTH SYSTEM RESEARCH SYMPOSIUM** or **5TH SINO FRENCH JOINT WORKSHOP ON ATMOSPHERIC ENVIRONMENT** or **6TH SINO FRENCH WORKSHOP ON ATMOSPHERIC ENVIRONMENT** or **7TH EUROPEAN SPORT ECONOMICS ASSOCIATION ESEA CONFERENCE ON SPORTS ECONOMICS** or **7TH INTERNATIONAL CONFERENCE ON FOG FOG COLLECTION AND DEW** or **8TH INTERNATIONAL CONGRESS OF NEUROENDOCRINOLOGY INTEGRATED WITH THE 18TH ANNUAL MEETING OF THE SOCIETY FOR BEHAVIOURAL NEUROENDOCRINOLOGY** or **ANNUAL MEETING OF THE AMERICAN COLLEGE OF SPORTS MEDICINE ACSM** or **ANNUAL MEETING OF THE EUROPEAN ACADEMY OF MANAGEMENT EURAM** or **CONFERENCE ON TACTICAL COMBAT CASUALTY CARE TRANSITIONING BATTLEFIELD LESSONS LEARNED TO OTHER AUSTERE ENVIRONMENTS IN CONJUNCTION WITH THE 7TH WORLD CONGRESS OF MOUNTAIN AND WILDERNESS MEDICINE** or **GEORGETOWN SYMPOSIUM ON GLOBAL MENTAL HEALTH TRANSDISCIPLINARY PERSPECTIVES** or **INTERNATIONAL BRAIN COMPUTER INTERFACE BCI MEETING** or **INTERNATIONAL CONFERENCE ON AGING AND DISEASE ICAD** or **ODLAA CONFERENCE** or **10TH ANNIVERSARY MEETING OF CONCEPTS ACTION AND OBJECTS CAOS** or **10TH ASIAN AEROSOL CONFERENCE AAC** or **10TH INTERNATIONAL CONFERENCE ON VIRTUAL REHABILITATION** or **10TH INTERNATIONAL MORPHOLOGICAL PROCESSING CONFERENCE MOPROC** or **11TH WORLD CONGRESS OF DEVELOPMENTAL ORIGINS OF HEALTH AND DISEASE DOHAD** or **12TH MEETING OF THE INTERNATIONAL BASAL GANGLIA SOCIETY IBAGS** or **13TH RESEARCH CENTERS IN MINORITY INSTITUTIONS RCMI INTERNATIONAL SYMPOSIUM ON HEALTH DISPARITIES** or **14TH BIENNIAL CONFERENCE OF THE SOUTH PACIFIC ENVIRONMENTAL RADIOACTIVITY ASSOCIATION SPERA** or **14TH CONFERENCE ON ENVIRONMENTAL PSYCHOLOGY PSICAMB** or **1ST WORLD CONGRESS ON GERIATRICS AND NEURODEGENERATIVE DISEASE RESEARCH GENEDIS** or **2014 NEUROBIOLOGY STRESS WORKSHOP** or **20TH INTERNATIONAL HYPOXIA SYMPOSIUM** or **20TH INTERNATIONAL SYMPOSIUM ON SHIFT WORK AND WORKING TIME** or **26TH NATIONAL CONFERENCE OF THE ITALIAN ASSOCIATION OF MEDICAL RADIATION PROTECTION AIRM** or **2ND ANNUAL MEETING OF THE CIC BIG TEN IVY LEAGUE TRAUMATIC BRAIN INJURY SUMMIT** or **2ND INTERNATIONAL CONFERENCE ON NATURAL AND ANTHROPIC RISKS** or **38TH ANNUAL MEETING OF THE AMERICAN ORTHOPAEDIC SOCIETY FOR SPORTS MEDICINE AOSSM** or **3RD ANNUAL ADDICTION DINNER AND SYMPOSIUM** or **3RD CONFERENCE ON INFORMATION INTEGRATION THEORY AND FUNCTIONAL MEASUREMENT** or **3RD FORUM ON THE NEUROBIOLOGY OF STRESS** or **3RD HIDDEN HUNGER CONFERENCE** or **3RD INTERNATIONAL CONFERENCE ON ENVIRONMENTAL MANAGEMENT ENGINEERING PLANNING AND ECONOMICS CEMEPE INTERNATIONAL SOCIETY FOR ECOTOXICOLOGY AND ENVIRONMENTAL SAFETY SECOTOX CONFERENCE** or **41ST ANNUAL MEETING OF THE AMERICAN ORTHOPAEDIC SOCIETY FOR SPORTS MEDICINE AOSSM** or **4TH ANNUAL WEIGHT AND STIGMA CONFERENCE** or **4TH CONFERENCE OF THE EUROPEAN SPORTS ECONOMICS ASSOCIATION ESEA ON SPORTS ECONOMICS** or **4TH INTERNATIONAL CONFERENCE ON LOGISTICS INFORMATICS AND SERVICE SCIENCE** or **5TH INTERNATIONAL SCIENTIFIC CONFERENCE ON MOTOR CONTROL** or **6TH ANIRCEF NATIONAL CONGRESS ON HEADACHE AND SOCIAL CONTEXT** or **6TH INTERNATIONAL SYMPOSIUM ON ECOLOGY AND ENVIRONMENTAL PROBLEMS** or **7TH ANNUAL CONFERENCE OF THE ORGANISATION FOR ONCOLOGY AND TRANSLATIONAL RESEARCH OOTR** or **7TH INTERNATIONAL PCB WORKSHOP CHEMICAL MIXTURES IN A COMPLEX WORLD** or **8TH INTERNATIONAL SYMPOSIUM ON RECENT ADVANCES IN ENVIRONMENTAL HEALTH RESEARCH** or **9TH INTERNATIONAL CONFERENCE ON THE ENVIRONMENTAL AND TECHNICAL IMPLICATIONS OF CONSTRUCTION WITH ALTERNATIVE MATERIALS WASCON RESOURCE EFFICIENCY IN CONSTRUCTION** or **9TH INTERNATIONAL MEETING ON STEROIDS AND NERVOUS SYSTEM** or **9TH WORLD CONGRESS ON CONTINUING PROFESSIONAL DEVELOPMENT CPD ADVANCING LEARNING AND CARE IN THE HEALTH PROFESSIONS** or **ANNUAL ASSEMBLY OF THE AMERICAN ACADEMY OF PHYSICAL MEDICINE AND REHABILITATION AAPM AND R** or **ANNUAL MEETING OF THE ASSOCIATION OF AMERICAN MEDICAL COLLEGES 54TH ANNUAL CONFERENCE ON RESEARCH IN MEDICAL EDUCATION** or **ANNUAL MEETING OF THE PAVLOVIAN SOCIETY** or **CONFERENCE ON ADVANCES IN MEDITATION RESEARCH AMR NEUROSCIENCE AND CLINICAL APPLICATIONS** or **CONFERENCE ON APPROACHING SYSTEMS BIOLOGY IN MARINE ANIMALS AND HUMANS** or **DAVID BARKER COMMEMORATIVE MEETING** or **GHRELIN SYMPOSIUM** or **INAUGURAL INTERNATIONAL CONFERENCE ON VESTIBULAR REHABILITATION ICVR** or **IUTAM SYMPOSIUM ON COMPLEXITY OF NONLINEAR WAVES** or **MEETING OF THE INTERNATIONAL BEHAVIORAL NEUROSCIENCE SOCIETY IBNS** or **NEWROSCIENCE INTERNATIONAL SYMPOSIUM** or **SIXTH INTERNATIONAL SYMPOSIUM OF SRC** or **TEACHER EDUCATION POLICY IN EUROPE TEPE** or **WINTER CONFERENCES OF SPORTS SCIENCE 6TH INTERNATIONAL CONGRESS AND 27TH NATIONAL CONGRESS OF PHYSICAL EDUCATION** or **WORLD CONFEDERATION FOR PHYSICAL THERAPY CONGRESS** or **11TH ASIAN AEROSOL CONFERENCE AAC** or **11TH CONFERENCE OF THE ASIAN SOCIETY FOR MITOCHONDRIAL RESEARCH AND MEDICINE FROM BENCH TO CLINIC ASMRM** or **12TH INTERNATIONAL CONFERENCE ON MYASTHENIA GRAVIS AND RELATED DISORDERS** or **12TH INTERNATIONAL CONGRESS OF INBORN ERRORS OF METABOLISM ICIEM** or **12TH SYMPOSIUM ON CATECHOLAMINES AND OTHER NEUROTRANSMITTERS IN STRESS** or **13TH FORUM OF THE INTERNATIONAL HCH AND PESTICIDES ASSOCIATION IHPA** or **13TH INTERNATIONAL CONFERENCE ON BRAIN ENERGY METABOLISM HOW METABOLISM DICTATES NEUROTRANSMISSION FUNCTION AND BEHAVIOR** or **13TH INTERNATIONAL CONFERENCE ON HARMONISATION WITHIN ATMOSPHERIC DISPERSION MODELLING FOR REGULATORY PURPOSES** or **13TH SAO PAULOS PUBLIC HEALTH CONFERENCE** or **14TH EUCHEMS INTERNATIONAL CONFERENCE ON CHEMISTRY AND THE ENVIRONMENT ICCE** or **15TH INTERNATIONAL SYMPOSIUM ON TOXICITY ASSESSMENT ISTA** or **17TH INTERNATIONAL SPORT SCIENCES CONGRESS** or **18TH INTERNATIONAL CONFERENCE ON HEAVY METALS IN THE ENVIRONMENT ICHMET** or **1ST INTERNATIONAL CONGRESS ON AUDIOGENIC EPILEPSY FROM MODELS TO THE CLINIC** or **2014 ANNUAL MEETING OF THE INTERNATIONAL BEHAVIORAL NEUROSCIENCE SOCIETY IBNS 2014** or **21ST WORLD CONGRESS OF GERONTOLOGY AND GERIATRICS OF THE INTERNATIONAL ASSOCIATION OF GERONTOLOGY AND GERIATRICS IAGG** or **23RD ANNUAL MEETING OF INTERNATIONAL BEHAVIORAL NEUROSCIENCE SOCIETY** or **23RD SRA EUROPE ANNUAL MEETING ON ANALYSIS AND GOVERNANCE OF RISKS BEYOND BOUNDARIES** or **24TH ANNUAL MEETING OF COGNITIVE NEUROSCIENCE SOCIETY CNS ON MULTIPLE NEUROCOMPUTATIONAL MOTIVATIONAL AND MNEMONIC MECHANISMS FOR DECISION MAKING** or **24TH ANNUAL MEETING OF COGNITIVE NEUROSCIENCE SOCIETY CNS ON REAL WORLD NEUROSCIENCE** or **24TH ANNUAL MEETING OF THE SOCIETY FOR TEXT AND DISCOURSE** or **26TH NATIONAL PHYSICAL MEDICINE AND REHABILITATION CONGRESS** or **28TH ANNUAL SCIENTIFIC MEETING OF THE STROKE SOCIETY OF AUSTRALASIA SSA** or **2ND CONFERENCE ON BIOETHICS ISSUES IN MINORITY HEALTH AND HEALTH DISPARITIES RESEARCH** or **2ND INTERNATIONAL CONFERENCE ON BIOLOGICAL WASTE AS RESOURCE BWR ENVIRONMENTAL IMPACT AND EMERGING CONCERNS OF BIOLOGICAL WASTE TREATMENT AND RECYCLING PROCESSES** or **2ND INTERNATIONAL CONFERENCE ON WATER ENERGY AND ENVIRONMENT ICWEE** or **36TH ANNUAL MEETING OF THE AMERICAN ORTHOPAEDIC SOCIETY FOR SPORTS MEDICINE AOSSM** or **3RD 3R INTERNATIONAL SCIENTIFIC CONFERENCE ON MATERIAL CYCLES AND WASTE MANAGEMENT 3RINCS** or **3RD BI ANNUAL MEETINGS ON SENSORY CORTICAL CIRCUITS WITH AN EMPHASIS ON THE BARREL CORTEX** or **3RD INTERNATIONAL CONFERENCE ON SUSTAINABLE SOLID WASTE MANAGEMENT** or **42ND ANNUAL MEETING OF THE AMERICAN ORTHOPAEDIC SOCIETY FOR SPORTS MEDICINE AOSSM** or **44TH ANNUAL MEETING OF THE SOCIETY FOR COMPUTERS IN PSYCHOLOGY SCIP** or **4TH INTERNATIONAL CONFERENCE ON FINAL SINKS ICFS** or **4TH INTERNATIONAL CONFERENCE ON UNDERSTANDING SMALL ENTERPRISES USE** or **4TH STEP CONFERENCE** or **4TH SYMPOSIUM OF FRENCH LANGUAGE DOHAD SOCIETY** or **4TH SYMPOSIUM ON PERSISTENT ORGANIC POLLUTANTS AND EMERGING CONTAMINANTS** or **5TH FLUX CONGRESS** or **5TH INTERNATIONAL MIND BRAIN AND EDUCATION SOCIETY IMBES CONFERENCE** or **6TH EUROPEAN SPORT ECONOMICS ASSOCIATION CONFERENCE** or **6TH IBERO AMERICAN CONGRESS ON QUALITATIVE RESEARCH CIAIQ 2ND INTERNATIONAL SYMPOSIUM ON QUALITATIVE RESEARCH ISQR** (Exclude – Conference Titles) and **Early Access** or **Book Chapters** or **Data Papers** or **Proceedings Papers** or **Retracted Publications** or **Publication With Expression Of Concern** or **Books** or **Withdrawn Publication** (Exclude – Document Types) and **Rct Or Ct** (Search within all fields)

# 16,143 results from Web of Science Core Collection
