# Supplementary material for: Modifiable determinants of older adults’ physical activity and sedentary behavior in community and healthcare settings: a DE-PASS systematic review and meta-analysis
Source: Eur Rev Aging Phys Act. 2025 May 24;22:9. doi: 10.1186/s11556-025-00373-y (PMC12103017; doi:10.1186/s11556-025-00373-y)
Supplement: Supplementary file 2 — Supplementary Material 2 [file 11556_2025_373_MOESM2_ESM.docx]

**Supplementary File 2.** Screening – Decision Tree

Does the population include older adults (aged 65 years and older)**^1^**?

Yes Maybe No -> Exclude (Click ‘No’)

Is physical activity (PAB) or sedentary behavior (SB) measured as an outcome**^2^** and at least two time points in the study?

Tag ‘PA or/and SB’

Yes Maybe No -> Exclude (Click ‘No’)

Is the study design**^3^** a Randomized Controlled Trial (RCT) or a Controlled Trial (CT)?

Tag ‘RCT’ or ‘CT’

Yes Maybe No -> Exclude (Click ‘No’)

Does the study only include participants without a dementia diagnosis or who are not currently being hospitalized and receiving medical treatment**^4^**?

Yes Maybe No-> Exclude (Click ‘No’)

Was the study performed outside the Covid-19 lockdown period?

Tag ‘Covid’

Yes Maybe No-> Exclude (Click ‘No’)

Are the determinants reported in the study modifiable AND measured**^5^**?

Yes Unsure No

Is the article available in English language?

Tag as ‘Non-English’

Yes No

INCLUDE (Click ‘Yes’) INCLUDE (Click ‘Maybe’)

**Definitions/Note**

**^1^ Age**

If a study includes participants both outside of and within our specified age span, e.g. 65 years and older, but mean age is ≥65.0 years, or information on participants in our specific age period (i.e. 65 years and older) is available to be extracted separately, then the study will be included. If the mean age is not available, or if there is no mentioning of whether or not information on our specific age span is presented/analyzed separately in the title/abstract, then the decision would be ‘Maybe’ in the title and abstract screening stage. If in the full text review stage the previously mentioned information/data are missing, then the study should be excluded. If the overall mean age is not reported in the full-text, but mean age of group is reported, the overall mean should be calculated using the following formula ((M1*N1) + (M2*N2)) /(N1+N2).

**^2^ PA/SB** **Outcome**

Measurement techniques for the assessment of PA and SB will encompass self-report (e.g., questionnaires, diaries, recall) and device-based (e.g., accelerometers, pedometers) methods.

PA/SB must be the outcome of a study. With regards to measurement, self-reports, proxy reports (e.g. from relatives) or device-based measures are considered eligible.

Studies that **ONLY** report symptoms (e.g., pain release) and physical performance (e.g., VO2 max, 6 m walk test, ADL) should not be included.

**^3^ Study designs**

A **randomized controlled trial (RCT)** involves randomly assigning 2 (or more) groups of participants to test the effectiveness of an intervention. One group (the experimental group) receives the intervention, the other group has an alternative intervention (the comparison group) or no intervention at all (control group). The groups are followed up to see how effective the intervention is.

A **controlled trial (CT)** is similar to RCT, involves assigning 2 (or more) groups of participants without (reported) randomization to test the effectiveness of an intervention. One group (the experimental group) receives the intervention, the other group has an alternative intervention (the comparison group) or no intervention at all (control group). Some studies might call their CTs quasi-experimental studies instead of controlled trials.

**Within subject studies** (e.g. one group pretest posttest design) WITHOUT A CONTROL GROUP are excluded.

**^4^** **Medical conditions**

Exclusion criteria encompasses:

1. The participants have a dementia diagnosis (including all dementia subtypes (e.g., Lewy body dementia, Alzheimer disease, Vascular dementia, Mixed dementia, Frontotemporal lobe dementia). Note that Mild Cognitive Impairment (MCI) and subjective memory complaints are **NOT** a dementia diagnosis.
2. The participants are currently hospitalized due to surgical or medical treatment (e.g cancer treatment).
3. The participants are included in pre- and post-operative studies on back, hip, knee, ankle joints, and bones of the thigh, leg, and foot.
4. The participants are receiving terminal or palliative care.

**^5^ Modifiable Determinants**

Determinant infers a potential causal relationship between a factor (e.g. motivation, motor skills, social support) and PA/SB outcomes. Some determinants are more modifiable than others. Examples of non-modifiable or least modifiable determinants include age, sex and genetics. You might also come across studies that have implemented interventions, e.g. changing institution facilities design, but no determinants have been measured. The intervention itself (called ‘intervention factor’) might be considered as a determinant in its own right, however, as there are no measurable determinants that tell us what about the institution facilities design that has got older adults more active (it could be to do with improving their motor skills, or the attractiveness of the space, or the facilities size), we cannot assess the effect of determinants on PA/SB. However, you may not know that these interventions have not measured any determinants until the full text review stage, as the measurement of determinants is not always mentioned in title/abstract. If this is the case, you should decide ‘Maybe’ at the title/abstract screening stage. If after full text review, there is indeed no information on the determinants measured, then tag the study as ‘Intervention factor only’ and click ‘No.
