# Supplementary material for: Modifiable determinants of older adults’ physical activity and sedentary behavior in community and healthcare settings: a DE-PASS systematic review and meta-analysis
Source: Eur Rev Aging Phys Act. 2025 May 24;22:9. doi: 10.1186/s11556-025-00373-y (PMC12103017; doi:10.1186/s11556-025-00373-y)

**Supplemenary File 3.** Forest plots of interventions without outliers.

*3A. Forest plot of interventions aimed at enhancing device-based physical activity by targeting physical health and wellbeing determinants without outliers
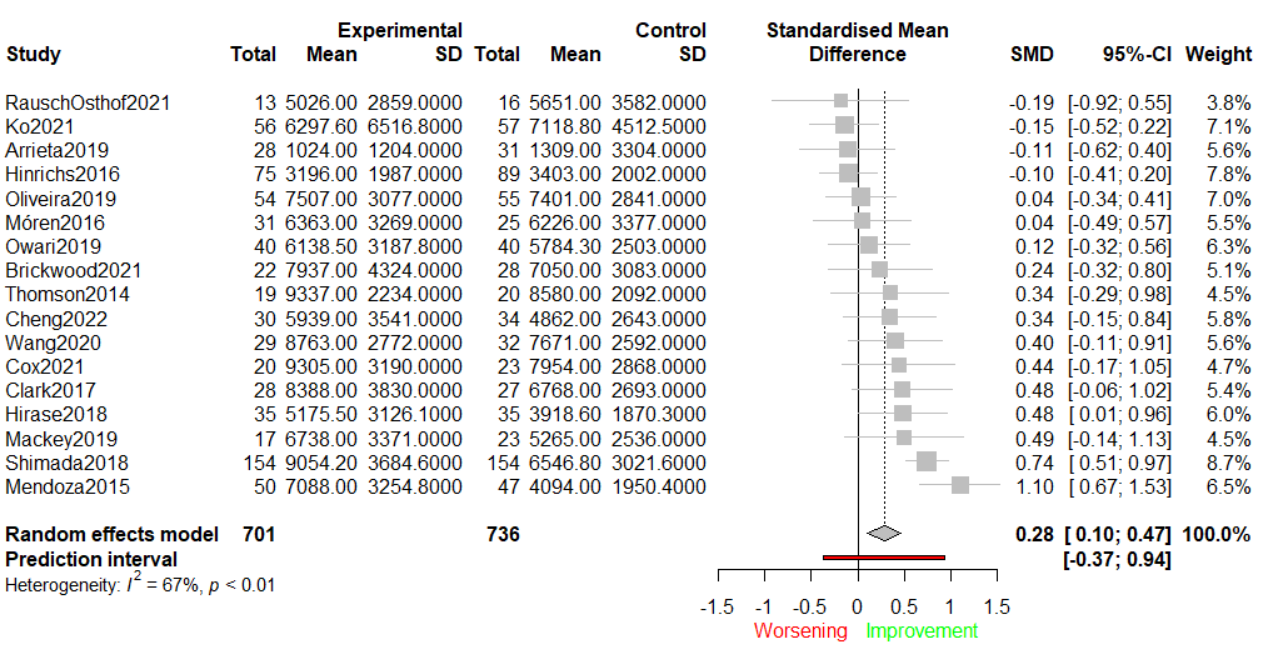
*

*3B. Forest plot* *of interventions aimed at enhancing device-based physical activity by targeting physical health and wellbeing determinants without low-quality studies*


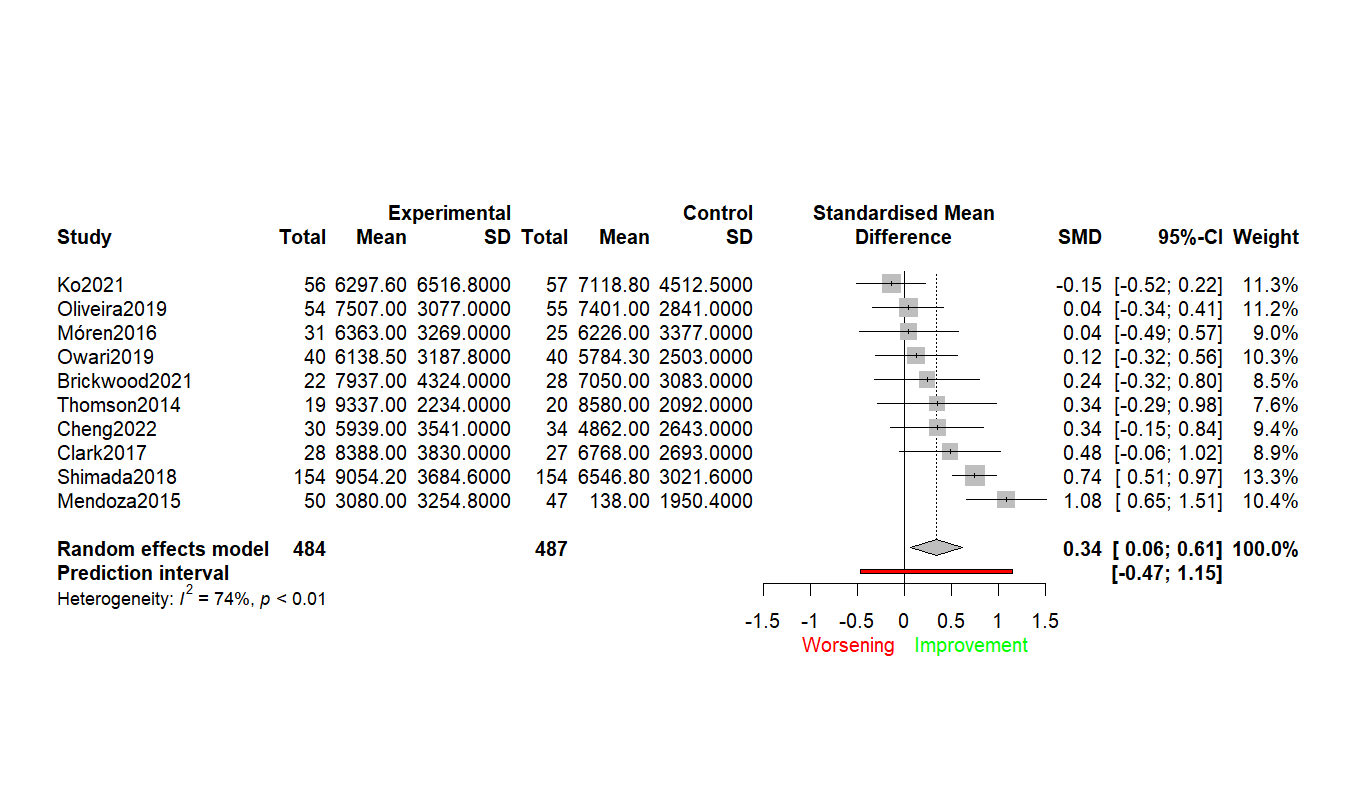

Supplement: Supplementary file 3 — Supplementary Material 3 [file 11556_2025_373_MOESM3_ESM.docx]
