# Supplementary material for: Modifiable determinants of older adults’ physical activity and sedentary behavior in community and healthcare settings: a DE-PASS systematic review and meta-analysis
Source: Eur Rev Aging Phys Act. 2025 May 24;22:9. doi: 10.1186/s11556-025-00373-y (PMC12103017; doi:10.1186/s11556-025-00373-y)

**Supplementary File 4.** Funnel plots of interventions.

*4A. Funnel plot of interventions aimed at enhancing device-based physical activity by targeting physical health and wellbeing determinants*


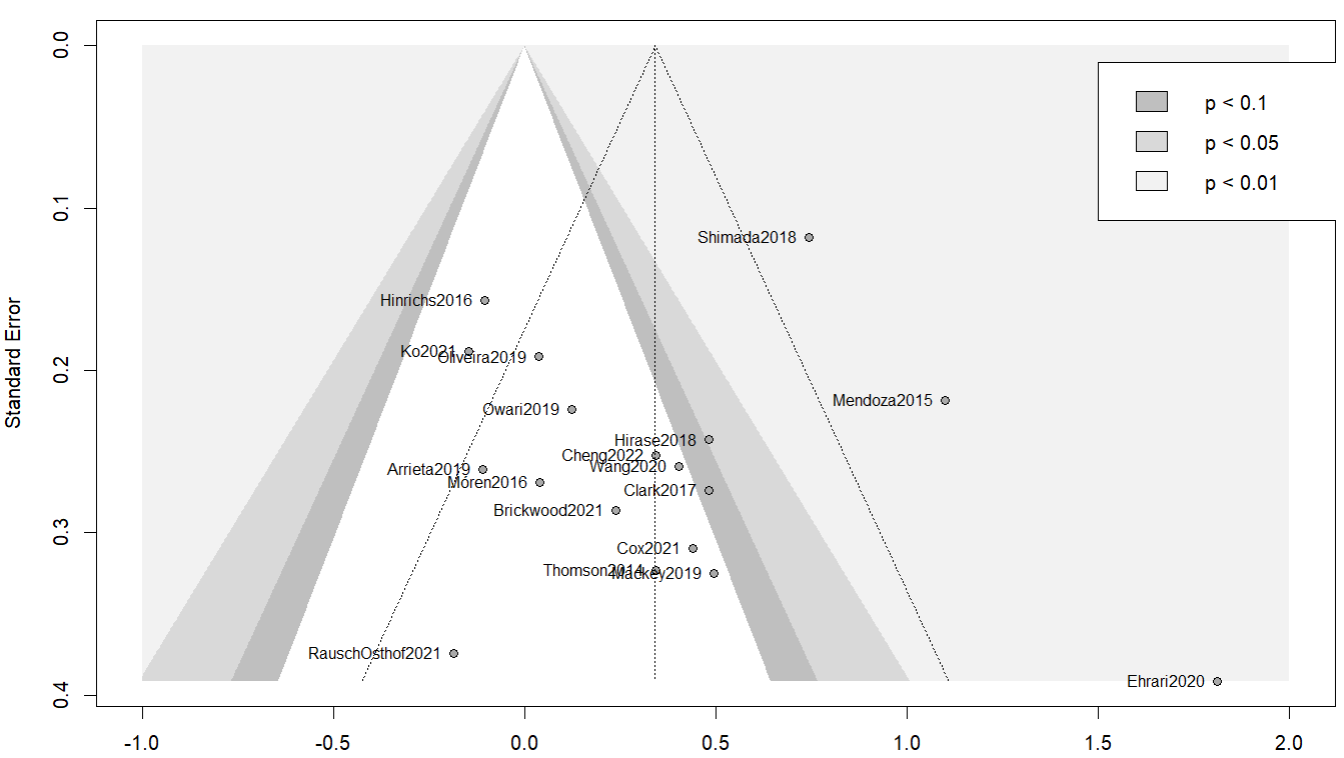


*4B. Funnel plot of interventions aimed at enhancing device-based physical activity by targeting psychological and behavioral determinants*

*
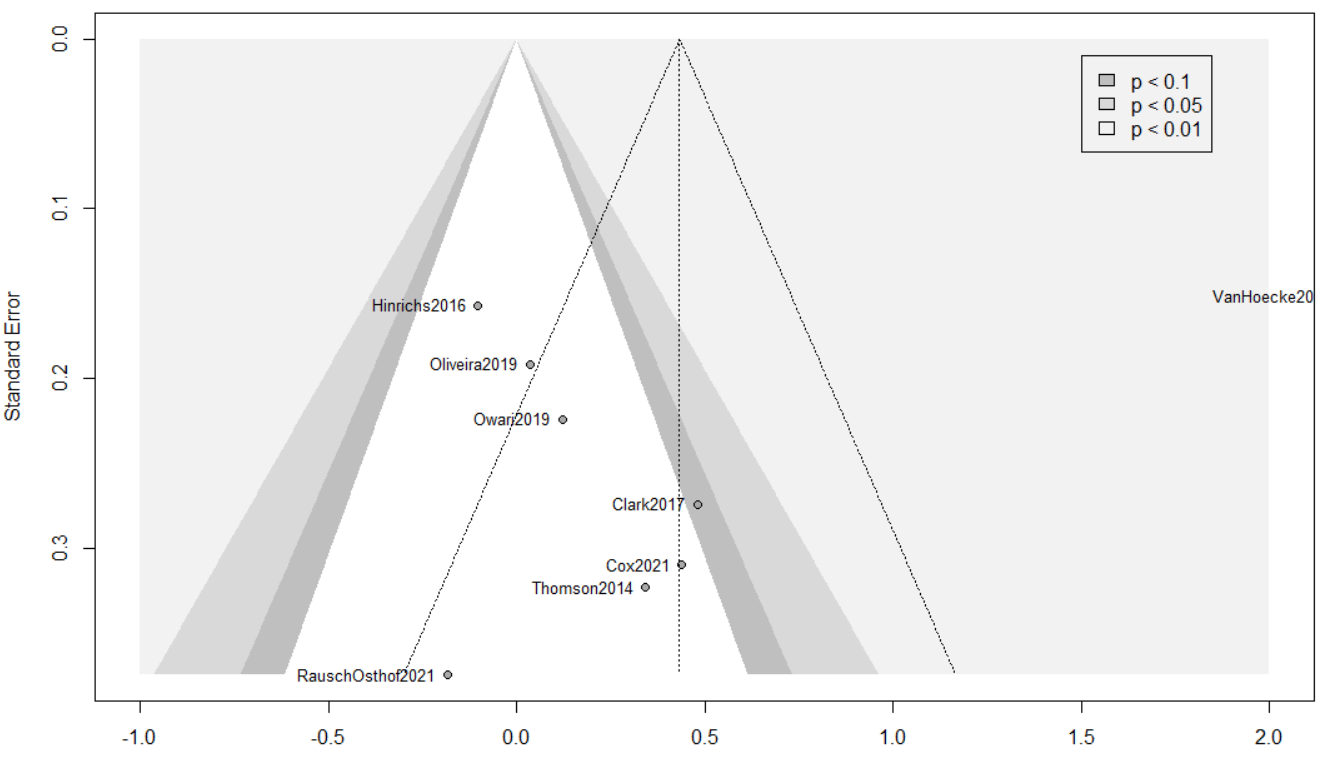
*

*4C. Funnel plot of interventions aimed at enhancing device-based sedentary behavior by targeting physical health and wellbeing determinants*


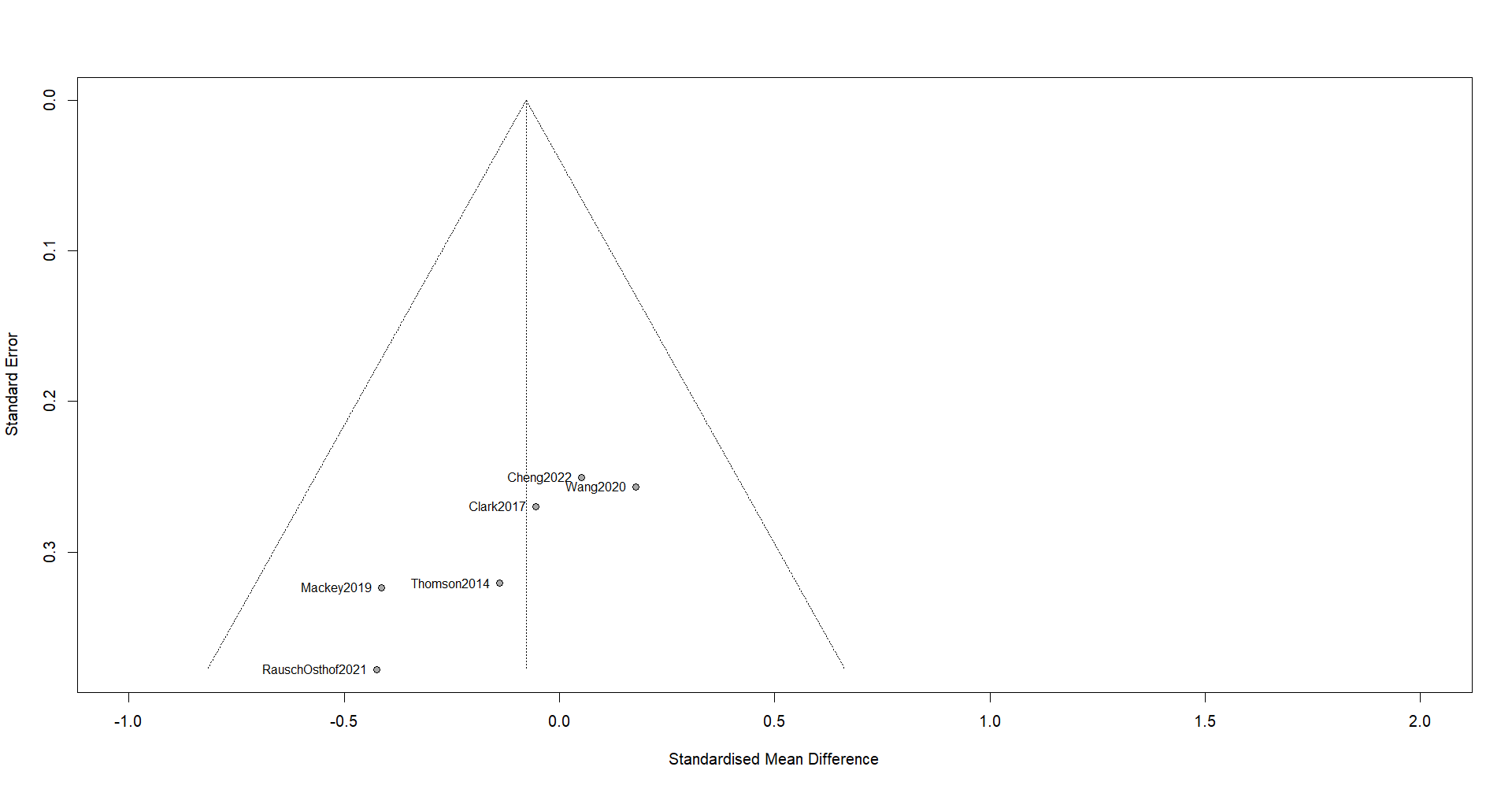


4D. *Funnel plot of interventions aimed at enhancing device-based sedentary behavior by targeting psychological and behavioral determinants*


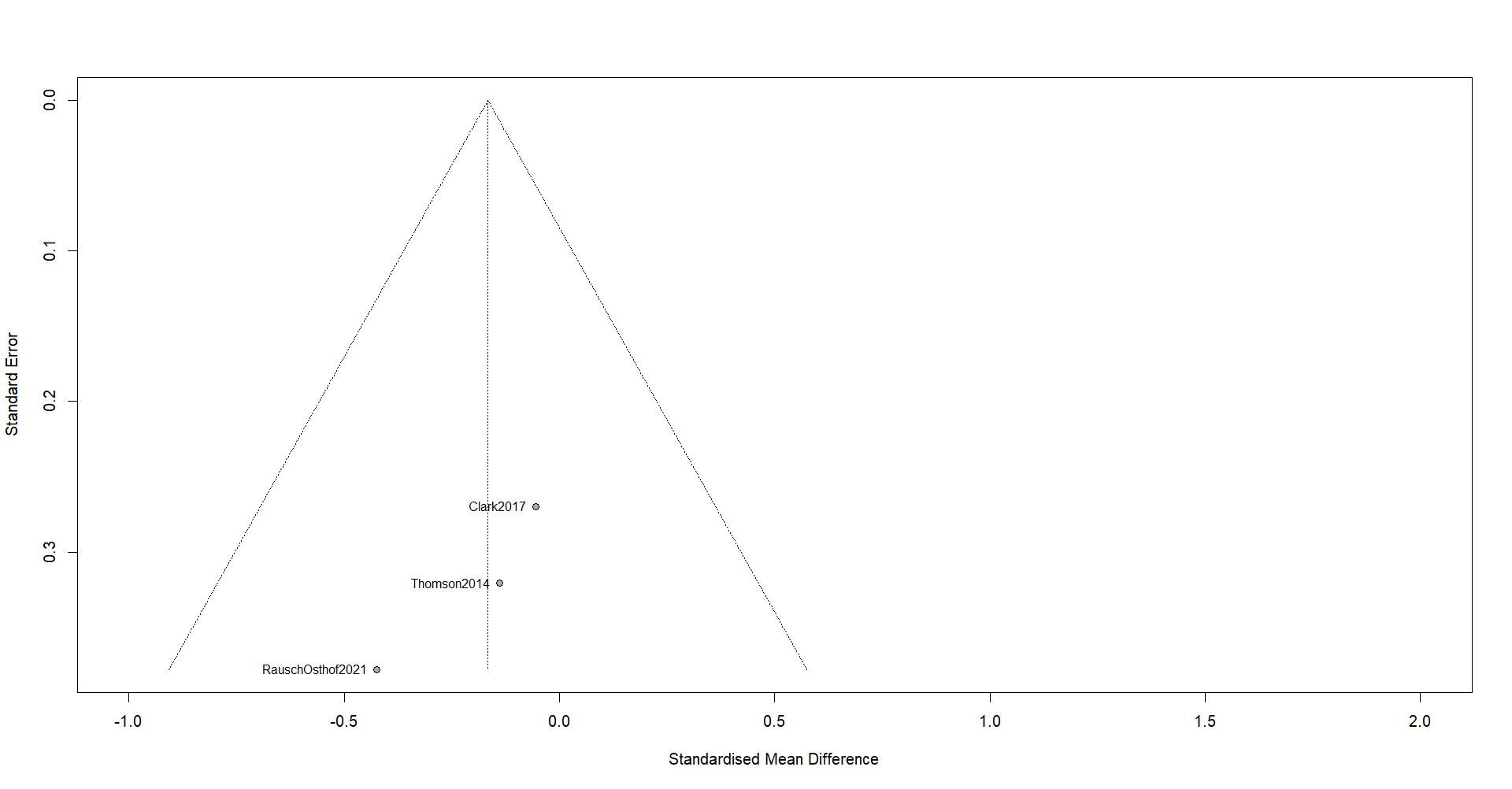

Supplement: Supplementary file 4 — Supplementary Material 4 [file 11556_2025_373_MOESM4_ESM.docx]
