# Supplementary material for: Modifiable determinants of older adults’ physical activity and sedentary behavior in community and healthcare settings: a DE-PASS systematic review and meta-analysis
Source: Eur Rev Aging Phys Act. 2025 May 24;22:9. doi: 10.1186/s11556-025-00373-y (PMC12103017; doi:10.1186/s11556-025-00373-y)
Supplement: Supplementary file 5 — Supplementary Material 5 [file 11556_2025_373_MOESM5_ESM.docx]

**Additional file 5.** Forest plots – intervention effects.

*Additional file 5A:* *Forest plot showing the effects of interventions targeting physical health and wellbeing determinants on MVPA*


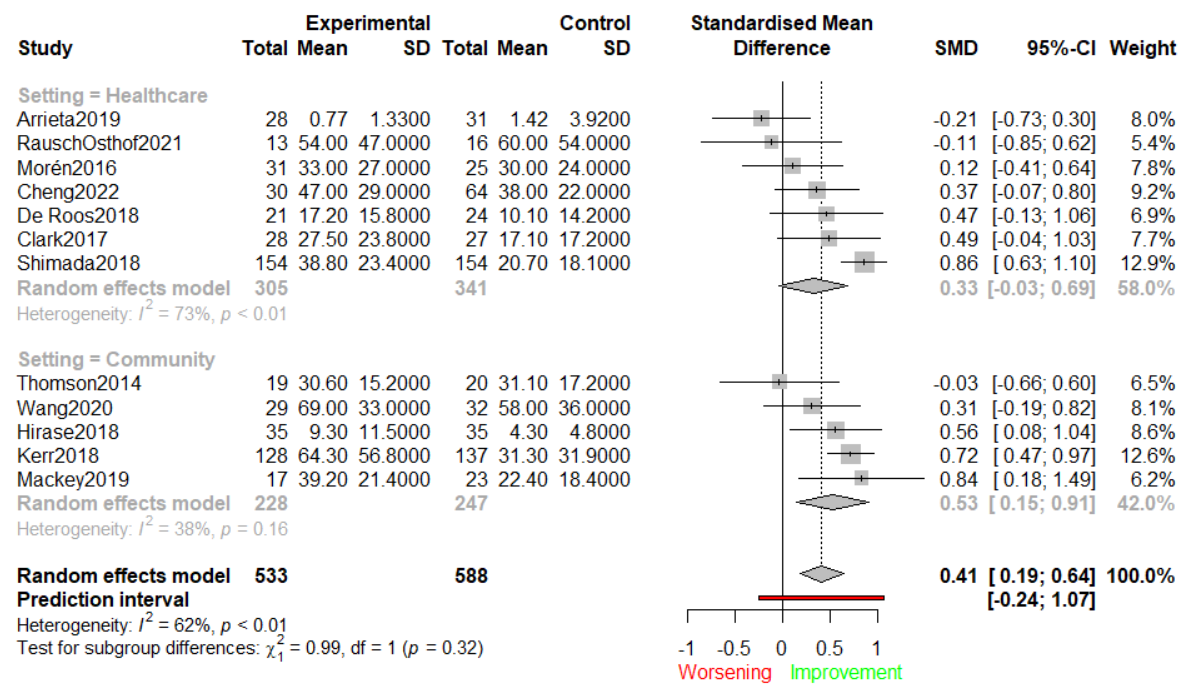


*Additional file 5B: Forest plot showing the effects of interventions targeting psychological and behavioral determinants on MVPA*

*
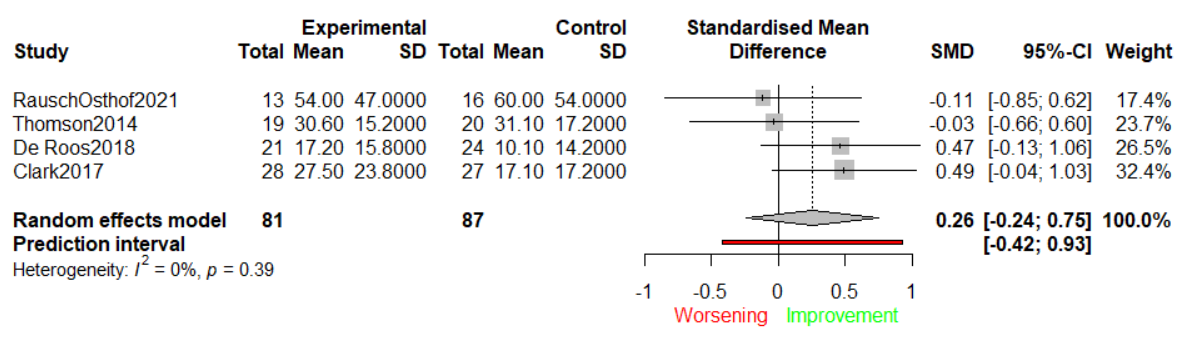
*
